# *Bacillus thuringiensis* PM25 ameliorates oxidative damage of salinity stress in maize *via* regulating growth, leaf pigments, antioxidant defense system, and stress responsive gene expression

**DOI:** 10.3389/fpls.2022.921668

**Published:** 2022-07-28

**Authors:** Baber Ali, Aqsa Hafeez, Saliha Ahmad, Muhammad Ammar Javed, Sumaira, Muhammad Siddique Afridi, Turki M. Dawoud, Khalid S. Almaary, Crina Carmen Muresan, Romina Alina Marc, Dalal Hussien M. Alkhalifah, Samy Selim

**Affiliations:** ^1^Department of Plant Sciences, Quaid-i-Azam University, Islamabad, Pakistan; ^2^Institute of Industrial Biotechnology, Government College University, Lahore, Pakistan; ^3^Department of Biotechnology, Quaid-i-Azam University, Islamabad, Pakistan; ^4^Department of Plant Pathology, Federal University of Lavras (UFLA), Lavras, MG, Brazil; ^5^Department of Botany and Microbiology, College of Science, King Saud University, Riyadh, Saudi Arabia; ^6^Food Engineering Department, Faculty of Food Science and Technology, University of Agricultural Science and Veterinary Medicine Cluj-Napoca, Cluj-Napoca, Romania; ^7^Department of Biology, College of Science, Princess Nourah Bint Abdulrahman University, Riyadh, Saudi Arabia; ^8^Department of Clinical Laboratory Sciences, College of Applied Medical Sciences, Jouf University, Sakaka, Saudi Arabia

**Keywords:** abiotic stress, antioxiants, qRT-PCR, plant-microbe interactions, PGPR—plant growth-promoting rhizobacteria

## Abstract

Soil salinity is the major abiotic stress that disrupts nutrient uptake, hinders plant growth, and threatens agricultural production. Plant growth-promoting rhizobacteria (PGPR) are the most promising eco-friendly beneficial microorganisms that can be used to improve plant responses against biotic and abiotic stresses. In this study, a previously identified *B. thuringiensis* PM25 showed tolerance to salinity stress up to 3 M NaCl. The Halo-tolerant *Bacillus thuringiensis* PM25 demonstrated distinct salinity tolerance and enhance plant growth-promoting activities under salinity stress. Antibiotic-resistant Iturin C (*ItuC*) and bio-surfactant-producing (*sfp* and *srfAA*) genes that confer biotic and abiotic stresses were also amplified in *B. thuringiensis* PM25. Under salinity stress, the physiological and molecular processes were followed by the over-expression of stress-related genes (APX and SOD) in *B. thuringiensis* PM25. The results detected that *B. thuringiensis* PM25 inoculation substantially improved phenotypic traits, chlorophyll content, radical scavenging capability, and relative water content under salinity stress. Under salinity stress, the inoculation of *B. thuringiensis* PM25 significantly increased antioxidant enzyme levels in inoculated maize as compared to uninoculated plants. In addition, *B. thuringiensis* PM25-inoculation dramatically increased soluble sugars, proteins, total phenols, and flavonoids in maize as compared to uninoculated plants. The inoculation of *B. thuringiensis* PM25 significantly reduced oxidative burst in inoculated maize under salinity stress, compared to uninoculated plants. Furthermore, *B. thuringiensis* PM25-inoculated plants had higher levels of compatible solutes than uninoculated controls. The current results demonstrated that *B. thuringiensis* PM25 plays an important role in reducing salinity stress by influencing antioxidant defense systems and abiotic stress-related genes. These findings also suggest that multi-stress tolerant *B. thuringiensis* PM25 could enhance plant growth by mitigating salt stress, which might be used as an innovative tool for enhancing plant yield and productivity.

## Introduction

Abiotic factors, such as cold, heat, drought (Wahab et al., 2022), heavy metals (Nawaz et al., 2022), salt concentration (Hussain et al., 2022), and nutrient deficiency (Ahmad et al., 2022) drastically affect crop yield and productivity. Salinity stress is the most detrimental threat to agricultural yield (Daliakopoulos et al., 2016). The high salt concentration of farming lands costs a loss of 27 billion US dollars annually (Singh et al., 2020). It affects about 20–33% of the irrigated and cultivated land worldwide, and by the year 2050, these pernicious effects are anticipated to rise to 50% (Machado and Serralheiro, 2017).

Excessive salt concentration significantly interferes with the plants' physiological functions, such as nitrogen fixation, ion homeostasis, lipid metabolism, photosynthesis, and protein synthesis (Li and Jiang, 2017). Salinity causes the excessive generation of toxic ions by producing water-deficient conditions, i.e., osmotic stress, culminating in producing toxic free radicals that foster oxidative damage (Islam et al., 2016). This osmotic stress results in delayed or poor germination and post-germination growth anomalies (Munns and Tester, 2008). The overabundance of ethylene and Na^+^ in high-salt stress inhibits macronutrient uptake and contrarily affects plant growth (Chen et al., 2007; Shibli et al., 2007). High salinity also fazes root growth by restricting plant cell division and elongation (Egamberdieva et al., 2017). Gaseous exchange, rate of transpiration, and photosynthesis are altered by infelicitous opening and closing of stomata under salinity stress. Furthermore, the uptake of necessary ions required for enzymatic and metabolic activities is also altered by high salt concentrations in soil (Hanin et al., 2016).

Certain reclamation techniques have been improvised to cope with excessive salt concentrations in soil. Some physical strategies, i.e., profile inversion, horizon mixing, deep plowing, channeling irrigation, sanding, and sub-soiling, can be implied, but their use is limited by their cost (Shrivastava and Kumar, 2015). The chemicals like gypsum, sulfur, and sulfuric acid can also be mixed with soil, but in dense soils, they can revert the reclamation process and increase salt concentration (Zia-ur-Rehman et al., 2016). The laboratory analysis and field applications have also supported electrodialysis, but the presence of insoluble compounds like CaSO_4_ limits its efficacy (Mosavat et al., 2012). Other than conventional methods, traditional plant breeding methods for introducing favorable traits into elite varieties have been used to produce stress-tolerant plants for a long time (Meena et al., 2017). The availability of genetic tools for salinity tolerance in crop plants, as well as reliable screening methods, recognition of genetic components of tolerance, and active genetic modification of required genetic backgrounds, are all crucial factors in breeding for inducing salinity tolerance in crop plants (Arzani and Ashraf, 2016). Similarly, genetic engineering approaches have been used to impart salt tolerance in plants. Despite all the promising studies, improving salt tolerance by engineering ion transport channels is a complicated and challenging task, particularly for improving salt tolerance while still requiring a high yield. To the best of our knowledge, no genetically modified (GM) crops with salt resistance have ever been commercialized (Fita et al., 2015). At the ground level, there has not been any success.

Plant growth-promoting rhizobacteria (PGPR) are the microorganisms that dwell around the plant roots establishing symbiotic relations and promoting the growth and development of the plant directly or indirectly. Numerous researchers have anticipated the role of PGPR in mitigating abiotic stress, especially their effectiveness in reducing the perilous effects of salt stress (Sharma et al., 2017). The implementation of useful PGPR in soil enhances plant growth, development, and salt stress tolerance through various mechanisms. They promulgate growth and stress tolerance in plants by accumulating osmolytes (OS), phosphate, and potassium solubilization, boosting nutrient uptake, nitrogen fixation, increasing water absorption capacity, siderophore sequestration, and enhancing antioxidant enzymes (AEs) activity (Islam et al., 2016; Ha-tran et al., 2021). They contain 1-aminoacyclopropane-1-carboxylic acid deaminase (ACCD), which curtails ethylene levels under salt stress by hydrolyzing ethylene precursor, which is 1-aminoacyclopropane-1-carboxylic acid (ACC) (Glick, 2014). These PGPR can also chelate excessive Na^+^ and minimize its availability by secreting exopolysaccharides (EPS) under salinity stress (Choudhary et al., 2016). Furthermore, they can also produce phytohormones, which alleviate the deleterious effects of saline stress (Dodd and Pérez-Alfocea, 2012). *Arthrobacter aurescens, Bacillus atrophaeus, Enterobacter asburiae*, and *Pseudomonas fluorescens* were the most effective plant growth-promoting bacteria (PGPB). These salt-tolerant rhizobia with plant growth-promoting traits would help wheat plants grow in saline conditions (Sharma et al., 2016).

Maize (*Zea mays* L.) is one of the most imperative cereal crops worldwide and the 3rd most cultivated crop after wheat and rice in Pakistan (Sharma et al., 2017). Maize is moderately susceptible to salinity stress, and Pakistan is the 8th largest salt-affected country (FAO, 2018). Therefore, alternative eco-friendly and significant strategies are needed to sustain crops in saline soils all over the globe. The application of plant growth-promoting rhizobacteria onto maize fields has shown substantial potential under saline conditions (Kumawat et al., 2022). These PGPR can mitigate the inimical effects of salt stress by modulating phytohormone regulations, enhancing proline content, nutrient acquisition, and antioxidant enzyme production, preventing transportation of salt ions into the plant tissues, and overexpression of stress-tolerant genes (Li et al., 2020; Mehmood et al., 2021b).

The hypothesis of the current study shows that *B. thuringiensis* PM25 mitigates the adverse effects of salinity stress on maize and reveals the physiological and molecular mechanisms of PM25-induced salinity stress tolerance in plants. This strain can be more scientifically applied as a biological inoculant to improve the productivity of crops in saline soils. This study investigated the PGP activities of rhizospheric bacteria, identified as *Bacillus thuringiensis* PM25, and its tolerance potential against salinity stress in maize plants. This study was aimed (i) to characterize *B. thuringiensis* PM25 based on its PGP traits and activity of extracellular enzymes; (ii) to assess the ability of *B. thuringiensis* PM25 to increase seedlings biomass under salinity stress, (iii) to reveal the impact of PGPR candidates on chlorophyll content, antioxidant enzyme activity, compatible solutes, oxidative stress markers, and osmolytes in bacterized *in-vitro*-grown maize supplemented with increasing NaCl concentrations, and (iv) to analyze the principal mechanisms of PGPR in plant growth promotion and salinity stress alleviation.

## Materials and Methods

### Acquirement of bacteria

All bacterial strains (PM21, PM22, PM23, PM26, PM27, PM28, B29, B30, B31, B32, B33, and B38) were obtained from Plant-Microbe Interactions Lab, Quaid-i-Azam University, Islamabad, Pakistan. The strains were evaluated against salinity tolerance potential (Afridi et al., 2019). *B. thuringiensis* PM25 showed the best salinity tolerance potential under different concentrations of NaCl (0, 1, 2, and 3 M). The *B. thuringiensis* PM25 sustained up to 3 M NaCl and grew significantly.

### Salinity tolerance characteristics of bacteria

The salinity tolerance of *B. thuringiensis* PM25 was estimated based on the population density under salinity stress (0, 300, 600, and 900 mM NaCl) in a tryptic soy broth (TSB) medium. Sterilized flasks containing 100 ml of TSB medium with different salt concentrations were used for analyzing the bacteria. The TSB medium was inoculated with 10 μl of freshly prepared bacterial broth of *B. thuringiensis* PM25 and incubated at 26 ± 2°C and 150 rpm in a shaking incubator. About 10 ml of sterilized broth with both salt concentrations was incubated as an uninoculated control. After 24 h of incubation, the optical density of the culture was measured at λ = 600 nm using a spectrophotometer (Agilent 8453 UV-visible Spectroscopy System), and the growth was compared with uninoculated control at a particular stress level (Afridi et al., 2019).

To estimate bacterial flocculation, *B. thuringiensis* PM25 was grown in a TSB medium with 0, 300, 600, and 900 mM NaCl for 72 h at 30°C. The flocculation was collected using Whatman No. 1 filter paper and oven-dried at 60°C. After 2 h, the dry weight of the floc yield was measured (Molina et al., 2021).

The bacterial strain *B. thuringiensis* PM25 was screened for sodium uptake capacity at different NaCl concentrations. The bacterium *B. thuringiensis* PM25 was grown overnight at 30°C in a TSB medium containing salinity stress (0, 300, 600, and 900 mM NaCl). The 24 h old bacterial cells were then centrifuged and harvested by centrifugation, and the bacterial pellet was washed with sterilized distilled water to remove the traces of medium. Before being digested overnight, the washed pellet was in 0.1 N HCl at room temperature. Centrifugation was performed to obtain the supernatant, and a flame photometer was used to assess bacterial sodium absorption (Shultana et al., 2020).

The biofilm-forming capacity of *B. thuringiensis* PM25 was quantitatively analyzed by measuring the number of cells attached to a glass disk incorporated within the Petri dish containing the bacterial culture under salinity stress (0, 300, 600, and 900 mM NaCl) (Prouty et al., 2002), using the crystal violet staining method proposed by O'Toole and Kolter (O'Toole and Kolter, 1998). The glass was taken under aseptic conditions at each exposure time, washed with 1 ml of NaCl (0.9% w/v), and treated with 1 ml of crystal violet indicator (0.1% w/v) over 20 min. Then, the glasses were washed three times with NaCl (0.9% w/v). Biofilm formation was quantified by adding 1 ml of 95% ethanol to each crystal violet stained glass. Biofilm was quantified at 506/570 nm using a UV spectrophotometer (752 N UV-VIS, Beijing, China).

### Estimation of plant growth-promoting traits

#### Indole-3-acetic acid production (IAA) production

The colorimetric method was followed to estimate (IAA) production (Loper and Schroth, 1986). A nutrient broth amended with 0.1% L-tryptophan and NaCl (0, 300, 600, and 900 mM) was inoculated with 1 ml of overnight-grown bacterial culture. The culture broth was incubated in the shaker at 180 rpm for 48 h in the dark at 28 ± 2°C. The bacterial culture was centrifuged at 10,000 rpm for 10 min at 4°C. The supernatant (1 ml) was mixed with 4 ml Salkowski reagent and the appearance of a pink color indicated the production of IAA (Gordon and Weber, 1951). The absorbance of the final pink color solution was measured after 30 min at 535 nm in a UV/Visible spectrophotometer and compared with the standard curve. The standard curve of IAA (Serva, Islandia, USA) was made in the range of 10–100 μg/ml to estimate IAA concentration.

#### Siderophore production

The screening of *B. thuriniensis* PM25 for siderophore production was performed using CAS (Chrome azurol S) agar media (Schwyn and Neilands, 1987). For the preparation of CAS agar, 60.5 mg of CAS was dissolved in 50 ml of distilled water. Furthermore, 10 ml of Fe^+3^ solution (1 mM FeCl_3_.6H_2_O) and 40 ml of HDTMA (Hexadecyl trimethylammonium bromide) (72.9 mg in 40 ml dH_2_O) were dissolved in a previously made CAS solution. Agar (15 g) was added to the resultant dark blue solution and was autoclaved. After the inoculation of the bacteria, plates were placed in an incubator for 7 d at 28°C. The development of orange zones around the bacterial inoculation revealed positive results for siderophore production. To estimate siderophore production, the protocol of Mehmood et al. (2021a) was followed. At 630 nm, optical density was observed and siderophore was estimated as percent siderophore unit (PSU) using the following formula:


$$\text{PSU }=\text{Ar}-\frac{\text{As}}{\text{Ar}}\;\times \;100$$

Where, As is inoculated sample absorbance and Ar is a reference (un-inoculated broth + CAS reagent + salt conc.).

#### 1-aminocyclopropane-1-carboxylate deaminase activity

The method of Zainab et al. (2021) was used to quantify the ACC deaminase. To quantify ACCD production by bacterial cultures, they were grown in Tryptic Soy Broth medium (TSB) for 24–48 h. The bacterial cells were harvested and centrifuged pellets were then washed with 0.1 M Tris HCl (pH = 7.5). The washed pellets were suspended in 2 ml of DF media containing 3 mM ACC supplemented with salinity stress (0, 300, 600, and 900 mM) and incubated in the cultures for 24–48 h again at 32°C. The bacterial cells were collected after 48 h by centrifugation at 300 rpm for 5 min, and pellets were washed with 2 ml of 0.1 M Tris HCl (pH = 7.5) and resuspended in 200 μl of 0.1 M Tris HCl (pH = 8.5). The bacterial pellets were labeled by adding 5% (v/v) toluen and then vortexed for 30 s. Then, 50 μl of each sample was incubated with 5 μl of 0.3 M ACC at 28°C for 30 min. Negative control had 50 μl of toluene labeled cells without ACC. Blank included 50 μl of toluene-labeled cells with 0.3 M 1-aminocyclopropane-1-carboxylate (ACC). Samples were mixed with 500 μl of 0.56 M HCl and centrifuged at 12,000 rpm for 5 min. Each 500 μl sample was taken from negative and blank in a glass test tube and added 400 μl of 0.56 N HCl, followed by 150 μl of 0.2% DNF solution and incubated for 30 min at 28°C. Before taking absorbance at 540 nm, 1 ml of 2 N NaOH was added. The activity was estimated by the hydrolysis of ACC into α- ketobutyrate. A standard curve of α- ketobutyrate was drawn ranging between 10 and 200 μmol and compared with absorbance taken at 540 nm of sample to determine μmol of α-ketobutyrate produced.

#### EPS production

The Exopolysaccharides quantification assay was performed following the method described by Ali et al. (2022a). The strain *B. thuringiensis* PM25 was grown in 50 ml ATCC no. 14-liquid medium: 0.2 g KH_2_PO_4_; 0.8 g K_2_HPO_4_; 0.2 g MgSO_4_ 0.7H_2_O; 0.1 g CaSO_4_ 0.2H_2_O; 2 mg FeCl_3_; Na 2MoO_4_ 0.2H_2_O (trace); 0.5 g yeast extract, 20 g sucrose; with pH of 7.2 by using sucrose as a carbon source (Ehsan et al., 2014) supplemented with NaCl (0, 300, 600, and 900 mM). The bacterium *B. thuringiensis* PM25 was also grown in 50 ml liquid medium ATCC no. 14 and incubated in a shaker for 3 days at 28°C with 200 rpm rotation. At the end of incubation, bacterial cells were harvested in pellet form by adding 1 mM EDTA, homogenized by shaking, and centrifuged for 10 min at 9,000 rpm. The supernatant containing EPS was separated and coupled with cold acetone with a proportion of 1:3. The mixture was again centrifuged at 15,000 rpm for 3 min. Deposition of EPS biomass was observed and washed with distilled water and dried until dry weights were fixed. The EPS was estimated as mg/mL of the dried weight.

### Soil analysis and seed inoculation

The soil was collected from the Quaid-i-Azam University, Islamabad, Pakistan (33.7470°N, 73.1371°E). The soil was first air-dried in the laboratory, and then it was crushed, sieved, and sterilized to get rid of all microbes and fungal spores (Afridi et al., 2019). The physio-chemical properties of the soil were assessed.

Certified maize seeds (SG-2002 variety) were collected from National Agricultural Research Center (NARC), Pakistan. Seeds were disinfected by serial washing with 70% ethyl alcohol for 5 min and 0.1% HgCl_2_ for 1 min. After disinfection, all seeds were rinsed three times with autoclaved distilled water. The strain *B. thuringiensis* PM25 was cultured in 250-ml flasks containing LB broth. After 48 h, the culture was taken and centrifuged for 10 min at 10,000 rpm to collect the pellet. The pellet was washed with 0.85% of NaCl, suspended in deionized water to maintain the absorbance at 0.5, and obtained a homogenous bacterial population [10^8^ colony-forming units (CFU) ml^−1^]. Seeds were dipped in bacterial solution for 2–4 h, while uninoculated seeds were soaked in sterilized water taken as control (Ali et al., 2022b).

### Experimental design

The Certified maize seeds (SG-2002 variety) were sown (6 surfaces of sterilized seeds per pot) in plastic pots containing 200 g of sterilized soil. Salinity stress was applied to the plants after 5 days of germination once a day with 80, 120, and 180 mM NaCl increments to the plants until reaching final concentrations of 300, 600, and 900 mM NaCl to avoid osmotic shock stress. The experimental design was summarized in Supplementary Table 1. Seeds were infected with bacteria and immersed in sterilized water for 2–4 h, whereas uninoculated seeds were used as a control.

The pots were placed in a growth chamber (CU-36L6, Iowa, USA) for 21 days. Each treatment received 20 ml of bacterial suspension after seed sowing within 7 days of the interval (7, 14 days), while the control was treated with 20 ml sterilized broth medium. Pots were rinsed with 50 ml of distilled water daily to maintain moisture for plant growth. Throughout the experiment, EC and the pH of the substrate in each pot were kept constant. The same quantity of water was sprayed regularly to maintain 60–70% of water holding capacity, balancing NaCl levels in each pot. Photosynthetic photon flux density (PPFD) levels were maintained up to 350 μmol. Humidity was maintained up to 60–80% in the growth chamber, the light duration for day and night was 12 h, and the temperature range was 32 and 20°C for day and night, respectively.

*Zea mays* L. plants were harvested, and their roots were wiped under running tap water after a 21 day pot experiment to eliminate soil particles from the root surface. The soil was removed from the roots, bagged plants, and taken to the lab for further testing.

### Estimation of phenotypic traits

After 21 days of the experiment, three randomly selected plants from each treatment and control were investigated for phenotypic traits, such as plant height, shoot, root length, and fresh and dry biomass. All plants were dried for 24 h in an 80°C hot air oven before being weighed. The total leaf area was calculated with the help of scale by using the formula L × B × K, where L denotes leaf length, B; leaf breadth, and K; Kemp's constant (For Monocot 0.9) (Shi et al., 2021).

### Estimation of photosynthetic pigments, relative water, and radical scavenging capacity of leaves

Photosynthetic pigments were extracted by homogenizing 0.1 g of fresh leaves with 6 ml of 80% ethanol. The extract was centrifuged, and the supernatant was taken in test tubes. A spectrophotometer (752 N UV-VIS, Beijing, China) was used to evaluate the optical density of chlorophyll *a, b*, and carotenoids at 663, 645, 510, and 480 nm, respectively (El-Esawi et al., 2018).


$$\begin{array}{l} \text{ Chlorophyll a }=(12.\text{7 }\times \text{ A}663)\;–\;(\text{2}.49\;\times \text{ A}645)\\ \text{ Chlorophyll b }=\;(12.9\;\times \text{ A}645)\;–\;(4.7\;\times \text{ A}663)\\ \text{Total chlorophyll }=\text{Chl a }+\text{ Chl b}\\ \text{ Carotenoids }=\;[(\text{7}.6\;\times \text{ OD480})\;–\text{ 1}.49(\text{OD}510)]\\ \;\times \;[(\text{Final volume of filtrate/1},000)\times \;0.5)] \end{array}$$

The relative water content (RWC) of green leaves was calculated by determining the turgid weight of fresh leaf samples and drying them in a hot air oven until they reached a consistent weight (Ali et al., 2021). A 0.5 g (FW) leaf was placed in a Petri dish filled with distilled water and left overnight in the dark. The turgid weight of the leaf was determined. After being heated at 72°C overnight, the leaf's dry weight (DW) was obtained.


$$\begin{array}{l} \text{RWC }\left(\text{\%}\right)\;=\;\left[\text{FW}-\text{DW}/\text{TW}-\text{DW}\right]\times 100 \end{array}$$

FW = Fresh Leaf weight; TW = Turgid leaf weight; DW = Dry leaf weight

Radical scavenging activity or 2, 2-diphenyl-1-picrylhydrazyl (DPPH) of the extracts was evaluated according to the protocol of Asgari et al. (2021). Fresh leaves (100 mg) were crushed in 80% methanol, centrifuged at 10,000 rpm, and collected the supernatant. A suitable volume of supernatant (2 ml) was mixed with 180 μl of DPPH (Aldrich Chemistry, Burlington, US) solution (0.1 mM). After 30 min, the mixture was discolored, and optical density (OD) was measured with a spectrophotometer (752 N UV-VIS, Beijing, China) at 517 nm.


$$\text{I }\left(\%\right)=\text{ Ac}-\frac{\text{As}}{\text{Ac}}\times 100$$

Where A_c_ = Control; A_s_ = Sample's absorbance

### Antioxidant enzymatic assays

Antioxidant activities (APX, SOD, and POD) were assessed in fresh leaves following the protocols of Hossain et al. (1984) and Afridi et al. (2019).

Fresh leaf samples (0.2 g) were crushed in a 2 ml extraction buffer (potassium phosphate, pH 7.5) and ascorbic acid (1 mM) to determine the APX level. The crushed materials were centrifuged for 20 min at 4°C and 13,000 rpm. The OD was obtained at 290 nm to evaluate APX. A standard curve was used to measure the activity in units/mg of proteins by estimating the decrement of ascorbate.

Using a precooled motor and pestle, freshly procured plant tissues (0.20 g) were crushed in 3 ml of 100 mM phosphate buffer (PB) for the POD assay. To separate the homogenate, the sample extract was centrifuged at 4°C and 10,000 rpm for 15 min. To determine peroxidase, an OD at 470 nm was obtained. One unit of POD is defined as the amount of enzyme that increases by 0.100 of absorbance at 436 nm/min.

To estimate SOD, the plant material was crushed in 4 ml of solution (1 g PVP, 0.0278 g Na_2_EDTA) and centrifuged at 10,000 rpm. A reaction mixture (400 μl H_2_O + 350 μl phosphate buffer + 100 μl methionine + 50 μl NBT + 50 μL enzyme extract + 50 μl riboflavin) was prepared to measure the activity of the SOD enzyme. The mixture was then exposed to light for 15 min, with the decrease in absorbance measured at 560 nm. A blank was made by omitting the enzyme extract. The activities of SOD were then calculated and expressed in milligrams per milligram of total soluble protein.

El-Saadony et al.'s (2021) methodology was used to assess the amount of ascorbic acid (AsA) in fresh leaves. The results were derived using an ascorbic acid (Sigma-Aldrich, St. Louis, USA) standard curve and expressed as mg/g FW.

### Total soluble sugars and proteins

The Grad technique was used to calculate total soluble sugars (TSS) (Grad et al., 2021). Fresh leaves (0.1 g) were homogenized with 3–5 ml 80% ethanol to eliminate all traces of soluble sugars and centrifuged for 10 min at 10,000 rpm. The supernatant was collected and processed to calculate TSS. A freshly prepared anthrone solution (3 ml) and 0.1 mL of alcoholic extract were mixed in test tubes. All test tubes were heated for 12 min in boiling water and then iced for 10 min before being incubated for 20 min at 25°C. The optical density of the solution was measured at 625 nm using a spectrophotometer (752 N UV-VIS, Beijing, China). The total soluble sugars were estimated in μg/mL of fresh weight using the glucose standard curve.

The protein content of fresh maize leaves was determined using Bovine Serum Albumin (BSA) (Sigma-Aldrich, St. Louis, MO, USA) as a reference, as described by Mendez and Kwon (2021). Fresh leaves (0.1 g) were crushed in a mortar and pestle with 1 ml of phosphate buffer (pH 7.5) and centrifuged for 10 min at 3,000 rpm. The total volume of supernatant (0.1 ml) in test tubes was increased to 1 ml by adding distilled water. Reagent C (Solution a and b in 50:1 ratio) (Solution a: 2% Na_2_CO_3_, 1% Na-K, 0.4% 0.1 N NaOH; Solution b: 0.5% CuSO_4_.5H_2_O in dH_2_O) (1 ml) was added, mixed for 10 min and then 0.1 ml of reagent D (Folin phenol: distill water in a 1:1 ratio) was added. Different concentrations (20, 40, 60, 80, 320, and 640 mg) of the BSA solution were prepared, then the absorbance of all samples was measured at 650 nm after 30 min of incubation.

### Total flavonoids and phenols

The total flavonoid content was determined using the aluminum chloride colorimetric assay (Woisky and Salatino, 1998). Fresh leaves (100 mg) were homogenized in 3 ml of 80% methanol for TFC estimation (Kim et al., 2005). In each tube, 0.50 ml of the extract was mixed with 1.50 ml of 95% ethanol and, 0.10 ml of 10% AlCl_3_. At room temperature, the absorbance was measured at 415 nm with a UV-Vis spectrophotometer (UV-9200, Beijing, China) after 30 min of incubation. The calibration curve was generated using quercetin (Sigma, St. Louis, USA). The quantitative evaluation was performed using a calibration curve with quercetin 1:1 (w/v) dissolved in absolute methanol as a reference and, results were computed in milligrams of Quercetin equivalents per 100 g fresh mass (mg QE/100).

The total phenolic content was determined spectrophotometrically using the Folin-phenolic Ciocalteau's reagent (Merck, Taufkirchen, Germany) (Tawaha et al., 2007). Folin-reagent (0.5 ml) and 0.45 ml of 7.5% (w/v) saturated sodium carbonate solution were added to methanol extracted samples (20 μl). After a 2-h incubation period at 25°C, the samples' absorbance was measured at 765 nm using a UV-VIS spectrophotometer (UV-9200, Beijing, China). Total phenolic compounds were computed and represented as mg gallic acid equivalent (mg GAE/100 g) sample using gallic acid (Sigma-Aldrich, St. Louis, USA) as a reference (100–800 mg/L). The absorbance at 750 nm of the reaction mixture was measured spectrophotometrically.

### Valuation of oxidative burst and osmolytes

The leakage of electrolytes from leaf disks was evaluated by determining the membrane stability index (Nisar et al., 2021). Leaf disks (0.10 g) of all treatments were placed in test tubes containing double distilled water. The EC of leaves was determined (C_1_) after 30 min in the water bath at 40°C. The same leaf sample was subsequently maintained in a water bath at 100°C for 10 min, and the EC has measured again (C_2_).


Membrane Stability Index = [1 – C1/C2] × 100

The methods of Li et al. (2013) and Kapoor et al. (2021) were followed to determine endogenous H_2_O_2_ and malondialdehyde (MDA) content, respectively. The fresh leaves (0.10 g) were homogenized with 3 m of 0.1% trichloroacetic acid (TCA) in an ice bath and centrifuged at 12,000 rpm for 15 min to determine H_2_O_2_ concentration. Then, 1 M potassium iodide (1 ml) and 10 mM potassium phosphate (0.50 m) buffer (pH 7.00) were added to the supernatant. The absorbance of the supernatant was measured (at 390 nm). On a standardized curve, the content of H_2_O_2_ was expressed.

In a cooled mortar and pestle containing 2 ml of 1% (w/v) trichloroacetic acid (TCA), a fresh leaf sample (0.2 g) was crushed. After centrifugation of 10 min at 15,000 rpm, 2 ml of the supernatant was removed and 4 ml of 0.5% thiobarbituric acid (TBA) was added to it. The mixture was heated to 95°C and then allowed to cool. The absorbance, at 532 and 600 nm, of all treated samples was determined. The quantity of TBA was calculated using the absorption coefficient of 1.55 mmol/cm.


$$\begin{array}{l} \text{MDA }=\text{Δ}\left(\text{OD}532-\text{OD}600\right)/1.56\times 10^{5} \end{array}$$

The ninhydrin method published by Shafiq et al. (2021) was used to determine free amino acids. The dried samples (200 mg) were homogenized in 5 ml of 80% alcohol and warmed for 15 min in a water bath. After that, the extract was centrifuged for 20 min at 2,000 rpm. In a water bath, a 0.20 ml sample of the reaction mixture was heated with 3.80 ml of ninhydrin reagent. The reaction mixture was cooled until it became purple-blue. At 570 nm, absorbance was measured. The standard curve was constructed using leucine amino acid and, findings were reported in mg of amino acid per gram of dry tissue.

The glycine betaine (GB) level was measured using a previously reported procedure (Luo et al., 2021). For glycine betaine quantification, the extract was made by homogenizing 500 mg of dried leaves with 5 ml distilled water and 0.05% toluene, placed for 24 h. The reaction mixture was filtered using 0.20-mm micropore filters before centrifuging for 5 min at 6,000 rpm. About 1 ml of HCl (2 N) and 0.10 ml of KI were stirred well with 0.50 ml of this extract. The mixture was chilled for 2 h and then violently agitated. Ice-cold water (2 ml) and 10 ml 1, 2-dichloroethane, or dichloromethane were carefully mixed with this extract. After removing the upper aqueous layer, the bottom, pink-colored layer was used to record optical density at 365 nm. The glycine betaine content in μg/gm dry weight was estimated through the betaine hydrochloride standard curve.

The method of Parveen and Siddiqui (2021) was utilized to determine proline content in shoots. Fresh shoot material (0.2 g) was crushed in 3 ml of 3% sulphosalicylic acid and stored at 5°C overnight. The obtained suspension was centrifuged for 5 min at 3,000 rpm. The supernatant (2 ml) was blended with an acidic ninhydrin reagent after centrifugation. This reagent was prepared by dissolving 1.25 g ninhydrin in 20 ml phosphoric acid (6 M) and 30 ml glacial acetic acid (1 M H_3_PO_4_ = 3 N H_3_PO_4_) with constant stirring. The reagent was kept stable for 24 h. The tubes carrying the contents were heated for 1 h in a water bath at 100°C. After cooling, the mixture was extracted with 4-ml toluene in a separate funnel. At 520 nm, optical density was determined using toluene as a blank.


$$\begin{array}{l} \text{Proline μg}/\text{g }=\text{ K}\times \text{DF}\times \text{Absorbance}/\text{FW} \end{array}$$

K = 17.52; Dilution factor = 2; Fresh weight = 0.5 g.

### Amplification of multi-stress related genes

The universal primers (Supplementary Table 2) were used to detect the Iturin C (*ItuC*) gene (Baysal et al., 2008). PCR reaction was done in a 25 μl reaction mixture. Thermal cycling conditions were initial activation at 95°C for 15 min, 35 cycles of 95°C for 1 min, 58°C for 1 min, and 72°C for 1.5 min, and at 72°C for 7 min.

The primers (Supplementary Table 2) were used for amplification of the *sfp* (Swaathy et al., 2014) and *srfAA* gene (Chung et al., 2008). The thermal cycler was set for an initial denaturation cycle of 1 min at 94°C, followed by 25 cycles of 1 min denaturation at 94°C, 30-sec annealing at 46°C, 1-min extension at 72°C, and a 10-min final extension at 72°C (Swaathy et al., 2014). The annealing temperature of the primer was adjusted to 58–60°C for the *srfAA* gene. The PCR result was examined using 2% of agarose gel during electrophoresis. The Gel Doc technique revealed distinct bands of the aforementioned genes.

### Antioxidant (APX and SOD) gene expression

The expression level of antioxidant genes (APX and SOD) was quantified by using quantitative real-time PCR (qRT-PCR) in the presence and absence of *B. thuriniensis* PM25 under salinity stress (0, 300, 600, and 900 mM NaCl). The Qiagen Rneasy Plant Mini kit (cat. Nos. 74903, Hilden, Germany) was used to isolate total RNA from maize plants and to remove the contaminated DNA. First-strand cDNA was synthesized using the Qiagen Reverse Transcription kit. The cDNA was then diluted to a final volume of 500 μl with ddH_2_O and real-time assays were performed with SYBR Green I Master (Roche, Basel, Switzerland) according to the manufacturer's recommendations. Reaction efficiency values were calculated by running each primer set on serial dilutions of a cDNA mixture comprising stressed and control plants. Real-time quantitative PCR (RT-qPCR) was carried out using the Qiagen Rneasy Plant Mini kit (cat. Nos. 74903, Hilden, Germany) with gene-specific primers designed for SOD (F: 5′-ACATTTGCTACCTCTCCCTCACCT-3′; R: 3′-TCGGGTAAGACATCGTCGGTATGT-5′), and APX (F: 5′-AAACCCAAGCTCAGAGAGCCTCAT-3′; R: 3′-TACTTCACGGTGCTTCTTGGTGGA-5′). Reaction mixture recipes were described in Supplementary Table 3. PCR amplification conditions were set up as described by Hu et al. (2018). PCRs were performed in a thermal cycler programmed as follows: an initial denaturing step at 95°C for 3 min, followed by 45 cycles of 94°C for 10 s, 20 s annealing for different primer at 50–55 and 72°C for 20 s, with a final elongation at 72°C for 5 min. The amplified products were applied to 1.2% (w/v) agarose gel and stained in ethidium bromide (0.5 μg ml^−1^). The strength of the fluorescent signal derived from ethidium bromide in each lane was determined by the Gel Doc system. The housekeeping gene Actin was used, and the amount of gene expression was measured using the 2^−ΔΔCt^ technique (Livak and Schmittgen, 2001).

### Statistical analysis

Experimental data was calculated by computing standard errors and means values. The Statistix 8.1 software was used to analyze all the collected data by applying analysis of variance (ANOVA) and a pairwise comparison between all mean values was computed by using the Least Significant Difference (LSD) test (*p* = 0.05). R-software was used for Principal Component Analysis (PCA) and Pearson correlation analysis for calculated data.

## Results

### Bacterial growth curve analysis

**Figure 1:**
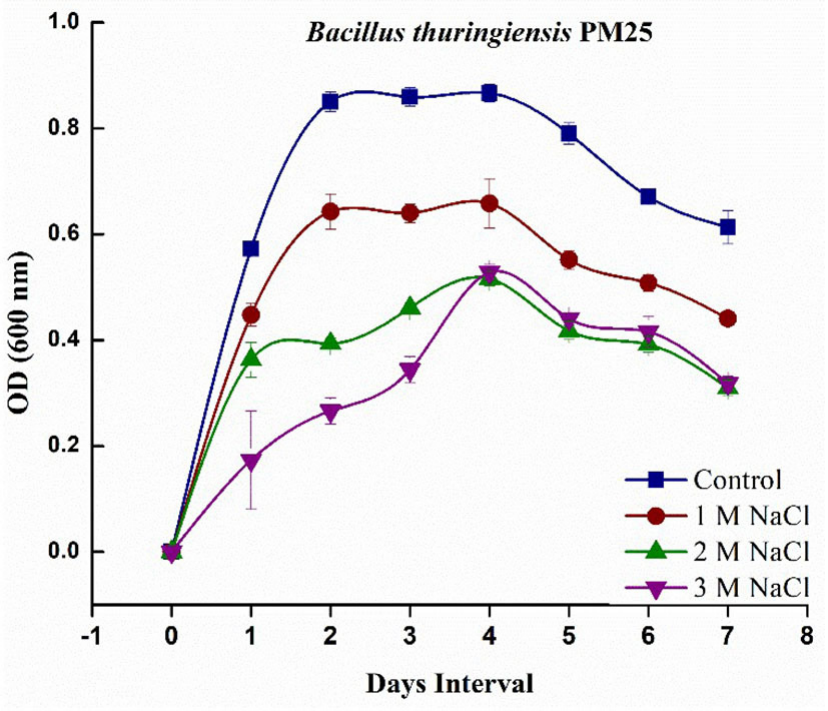
Growth curve analysis of *Bacillus thuringiensis* PM25 under salinity stress (0, 1, 2, and 3 M NaCl).

Salinity negatively affects biological activity by high osmotic strength (low water potential), which can be attributed to the toxic effect on microbial growth, except for tolerant halophytic bacteria. The salinity tolerance potential of *Bacillus thuringiensis* PM25 revealed its ability to grow in various salt concentrations applied (0, 1, 2, and 3 M). It was investigated that in Luria-Bertani (LB) medium*, B. thuringiensis* PM25 could tolerate salt stress up to 3 M NaCl concentration. A log phase is revealed on the 4th day of incubation in growth curve analysis (Figure 1).

### Salinity tolerance characteristics of bacteria

#### Bacterial survivability

**Figure 2:**
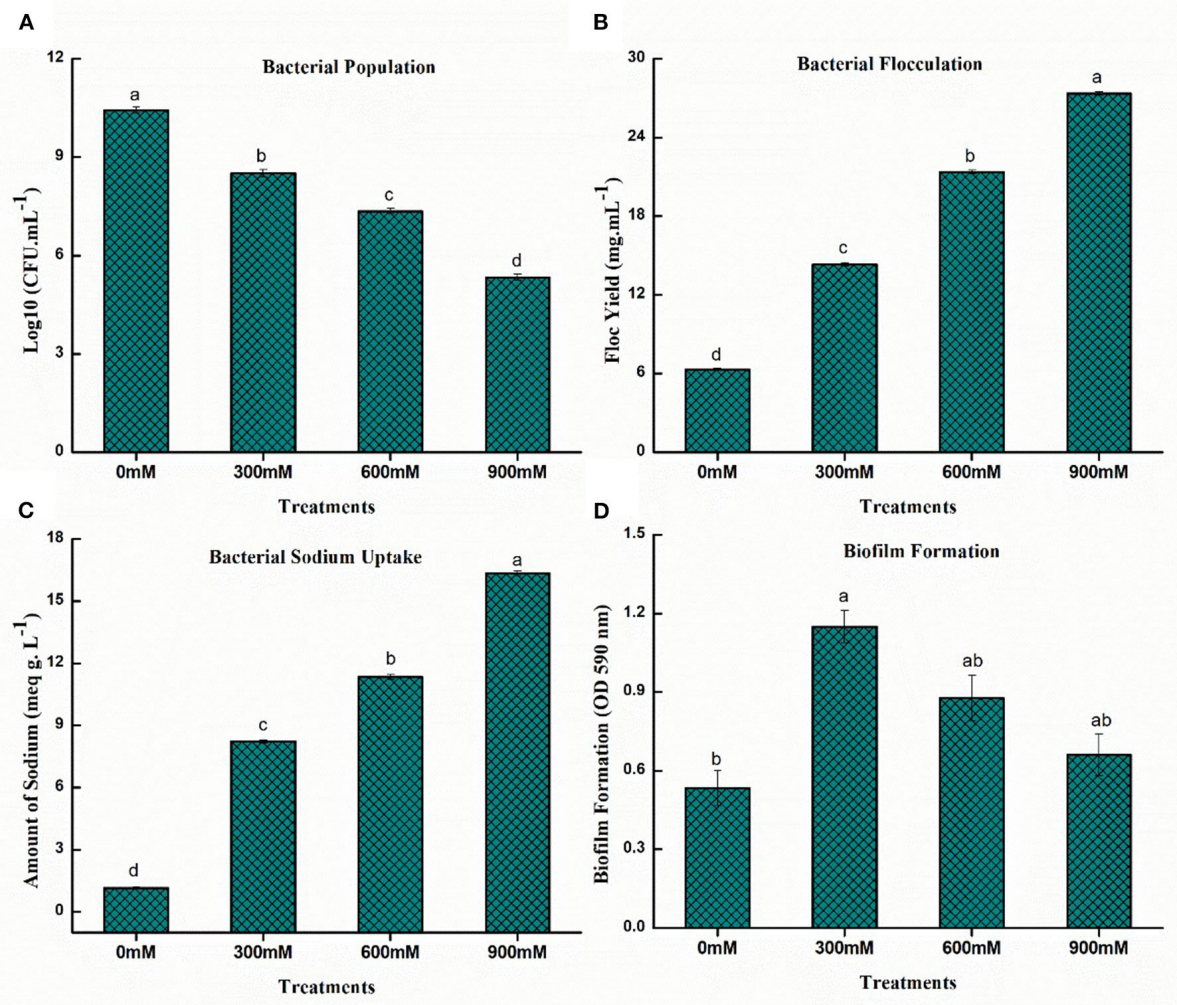
Effects of NaCl on salinity tolerance traits of PM25 **(A)** Bacterial population **(B)** Flocculation yield **(C)** Bacterial Na+ uptake **(D)** Biofilm formation. Bars sharing different letter (s) for each parameter are significantly different from each other according to the Least Significant Difference (LSD) test (*p* ≤ 0.05). All the data represented are the average of three replications (*n* = 3). Error bars represent the standard errors (SE) of three replicates.

The bacterial population in the culture medium plate was calculated with the number of colony-forming units (CFUs). The bacterial population was inversely proportional to salinity stress and a substantial drop in population (48.80%) of *B. thuringiensis* PM25 was observed at 900 mM NaCl while compared to control (Figure 2A).

#### Bacterial flocculation

In the case of bacterial flocculation yield, a direct relation was observed under salinity stress (Figure 2B). The flocculation yield increased significantly (76.45%) at 900 mM NaCl while compared to control (Figure 2B).

#### Bacterial sodium uptake

Accumulation of EPS-producing bacteria in the root zone of the plant reduces the accessibility of Na^+^ ions in the rhizospheric region and alleviates salinity stress. The sodium absorption of *B. thuringiensis* PM25 followed a similar pattern as floc yield. The Na^+^ uptake was directly proportional to the increasing salinity stress (Figure 2C). The bacterial sodium uptake was significantly increased (93.01%) at 900 mM NaCl while compared to control (Figure 2C).

#### Biofilm formation

Salt-tolerant halophilic bacteria mainly form biofilms that confer salt-stress resistance. Biofilms contain exopolysaccharides (EPSs), which can protect bacterial cells against high salinity. The production of biofilm is specifically correlated to the production of EPS. The biofilm production *of B. thuringiensis* PM25 was significantly increased (19.69%) at 900 mM NaCl while compared to control (Figure 2D).

### Quantification of PGP traits of bacteria

**Figure 3:**
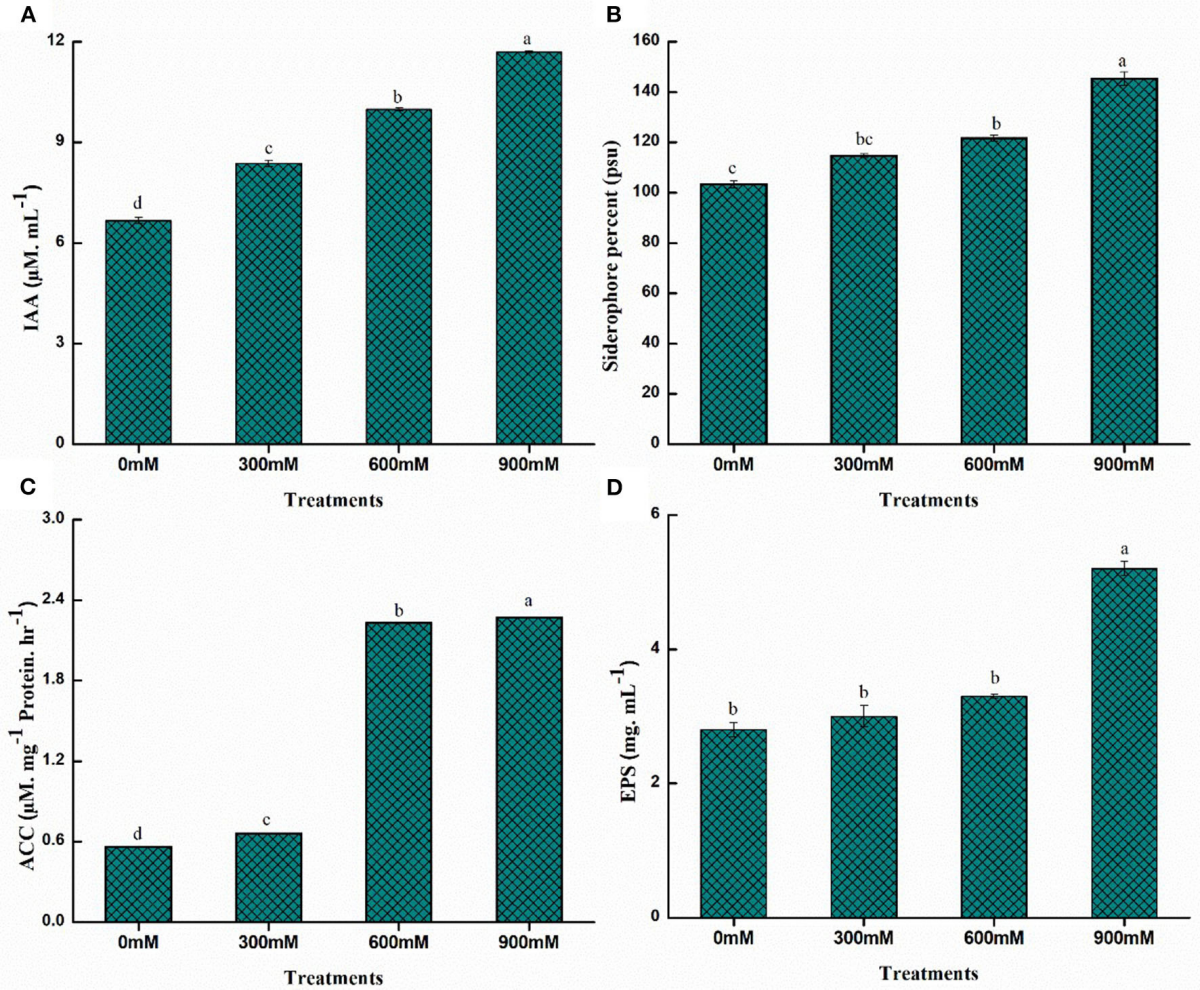
Quantitative estimation of PGP traits of PM25 under salinity stress: **(A)** IAA **(B)** Siderophore **(C)** ACCD **(D)** EPS. Bars sharing different letter (s) for each parameter are significantly different from each other according to the LSD test (*p* ≤ 0.05). All the data represented are the average of three replications (*n* = 3). Error bars represent the standard errors (SE) of three replicates.

The PGPR exhibits beneficial traits in mitigating the toxic effects of high salt concentrations on morphological, physiological, and biochemical processes in plants, resulting in the significant rescue of yield loss. PGPR mitigates salinity stress symptoms by producing exopolysaccharides (EPS), improving ion homeostasis, decreasing ethylene levels through enzyme 1-aminocyclopropane-1-carboxylate (ACC) deaminase, and synthesizing phytohormones. The quantitative estimation of PGP traits for *B. thuringiensis* PM25 revealed increased production with increasing salinity stress (Figure 3). Significantly higher values were observed for all PGP traits under salinity stress (900 mM NaCl). Maximum increment for IAA, siderophore, ACC deaminase, and EPS were 43.02, 42, 75.33, and 46.15%, respectively, at 900 mM NaCl while comparing to control (Figure 3).

### Pre-sowing and post-harvesting soil analysis

Table 1 shows the physicochemical parameters of soil. Pre-sowing and Post-harvesting soils had a loamy texture and mild alkalinity, with electrical conductivities of 1.53 and 4.49 dS/m, respectively. In pre-sowing soil, there was more organic matter and saturation than in post-harvested soil. Similarly, pre-sowing soil had higher phosphate and potassium levels than post-harvested soil (Table 1).

**Table 1:** Physio-chemical properties of soil.

**Soil parameters**	**Soil 1 (pre-sowing)**	**Soil 2 (post-harvesting)**
Soil texture	Loamy	Loamy
pH	7.94	7.87
Electrical conductivity (dS/m)	1.53	4.49
Organic matter (%)	3.49	1.88
Available phosphorus (mg/kg)	45.62	33.85
Available potassium (mg/kg)	601	124
Saturation (%)	44	43

### Pot experiment under controlled conditions

A pot experiment was conducted for 21 days, and maize plants were then carefully harvested. The experiment evaluated the effect of *B. thuringiensis* PM25, as a halotolerant PGP strain, on growth parameters of *Zea mays* L.

### Phenotypic traits of maize

High salinity significantly affects plant growth and physio-biochemical aspects, resulting in a decrease in germination rate (GRA), fresh and dry matters, photosynthetic pigments, essential nutrients uptake, and, most importantly, the loss of final crop yields. The co-cultivation of plant growth-promoting bacteria and plants in saline soils may reduce the severity of salinity stress, together with enhancing plant growth and crop yield. Before the application of *B. thuringiensis* PM25, the effect of salinity stress was determined. A significant reduction in shoot and root length was observed (Table 2). Plants that had been injected with bacteria, on the other hand, had longer shoots and roots than plants that had not been inoculated (Table 2). The results demonstrated that inoculating maize plants with *B. thuringiensis* PM25 had promising effects on all metrics under normal and salt stress conditions from 300 to 900 mM NaCl. By treating maize plants with *B. thuringiensis* PM25 under salinity stress, the shoot (17–41%), root length (33–48%), plant height (23–41%), fresh weight (55–69%), dry weight (53–59%), and leaf surface area (26–34%) grew significantly as compared to respective controls (Tables 2, 3). With and without salt stress, the PM25-inoculated plant showed an increase in all parameters, resulting in enhanced growth and biomass (Tables 2, 3).

**Table 2:** Maize growth, biomass, and leaf surface area in the presence and absence of *Bacillus thuringiensis* PM25 under salinity stress.

**NaCl (mM)**	***B. thuringiensis*** **PM25**	**Shoot length (cm)**	**Root length (cm)**	**Plant height (cm)**	**Leaf area (cm** ^2^ **)**
0 mM	–PM25	31.33 ± 1.25^b^	13.36 ± 0.60^bcd^	44.7 ± 0.68^bc^	16.01 ± 1.11^bc^
	+PM25	38 ± 1.34^a^	20 ± 1.66^a^	58 ± 2.93^a^	23.55 ± 1.30^a^
300 mM	–PM25	28.83 ± 0.88^bc^	10 ± 0.53^cde^	38.83 ± 1.40^cd^	14.89 ± 0.82^bcd^
	+PM25	34.73 ± 1.23^ab^	16.5 ± 1.30^ab^	51.23 ± 1.41^ab^	20.16 ± 1.24^ab^
600 mM	–PM25	24.53 ± 0.46^c^	8.07 ± 0.50^de^	32.60 ± 0.78^de^	12.13 ± 0.53^cd^
	+PM25	32.33 ± 0.30^ab^	15.51 ± 0.73^abc^	47.85 ± 1.02^abc^	16.75 ± 1.05^bc^
900 mM	–PM25	17.26 ± 0.69^d^	6.13 ± 0.39^e^	23.4 ± 1.07^e^	9.02 ± 0.60^d^
	+PM25	29.33 ± 1.36^bc^	10 ± 1.06^cde^	39.33 ± 1.96^cd^	13.63 ± 0.82^cd^

*Growth was measured at 21 days after seed owing under different salt concentration regimes. The treatments exhibit dissimilar letters within rows that represent a significant (p ≤ 0.05) level*.

**Table 3:** Biomass, leaf surface area, relative water content, and antioxidant activity of maize in the presence and absence of *Bacillus thuringiensis* PM25 under salinity stress.

**NaCl (mM)**	***B. thuringiensis*** **PM25**	**Fresh weight (g)**	**Dry weight (g)**	**RWC (%)**	**DPPH (IC**_50_ **) %**
0 mM	–PM25	1.14 ± 0.16^bcd^	0.37 ± 0.06^bc^	47.76 ± 0.74^bc^	35.14 ± 1.63^e^
	+PM25	3.22 ± 0.08^a^	0.78 ± 0.05^a^	61.58 ± 1.01^a^	45.15 ± 1.16^cd^
300 mM	–PM25	0.80 ± 0.12^cd^	0.31 ± 0.06^bc^	43.39 ± 1.17^cd^	38.48 ± 1.91^de^
	+PM25	1.78 ± 0.20^b^	0.71 ± 0.04^a^	58.82 ± 0.71^a^	49.34 ± 1.00^bc^
600 mM	–PM25	0.57 ± 0.09^d^	0.27 ± 0.05^bc^	37.88 ± 0.95^de^	43.30 ± 0.85^cd^
	+PM25	1.75 ± 0.05^b^	0.59 ± 0.05^ab^	52.51 ± 0.79^b^	53.33 ± 0.27^b^
900 mM	–PM25	0.46 ± 0.07^b^	0.21 ± 0.02^c^	34.22 ± 0.79^e^	48.41 ± 0.50^bc^
	+PM25	1.49 ± 0.04^bc^	0.51 ± 0.03^abc^	47.95 ± 0.81^bc^	60.63 ± 0.20^a^

*Growth was measured at 21 days after seed owing under different salt concentration regimes. RWC–Relative water content, DPPH–Radical scavenging activity of leaves. The treatments exhibit dissimilar letters within rows that represent a significant (p ≤ 0.05) level*.

### Relative water content and DPPH activity of maize

Bacterial EPS and biofilm have promising water retention capacity. Therefore, EPS-producing PGPR shows significant tolerance to abiotic stress like drought and salinity. Leaves of maize plants were further subjected to the analysis for RWC under salinity stress and results showed a significant decrease, along with increasing concentrations of NaCl (Table 3). Under control conditions (0 mM), the *B. thuringiensis* PM25 inoculated maize plants have shown a rise in RWC. Under salinity stress, a significantly higher RWC was noted at 600 mM (28%) and 900 mM (29%) than 300 mM NaCl (26%) in inoculated maize plants as compared to the respective control (Table 3). The radical scavenging capacity was analyzed at 0, 300, 600, and 900 mM NaCl in *B. thuringiensis* PM25 inoculated and un-inoculated plants (Table 3). Under salinity stress, PM25-inoculated plants had greater DPPH than uninoculated plants. However, the increment was significant (20%) at 900 mM NaCl compared to the control (Table 3).

### Estimation of photosynthetic pigments of plants

Salinity stress causes unrepairable damage to the photosynthetic apparatus at any development stage of a plant's life as it alters the structure of the chloroplasts, degrades the chloroplast envelope, and triggers chloroplast protrusions. Three different treatments were designed to analyze the positive effect of *B. thuringiensis* PM25 on pigments (Table 4). This experiment showed that salinity stress causes a negative effect on all photosynthetic pigments. The amount of pigmented content decreased under salinity stress (Table 4). The *B. thuringiensis* PM25-inoculated plants revealed a positive effect on pigments as compared to un-inoculated counterparts. The more promising increase was observed in chl *a* (33%), chl *b* (58%), total chlorophyll (42%), and carotenoids (53%) by inoculation of *B. thuringiensis* PM25 under salinity stress (900 mM NaCl) while comparing to respective control (Table 4).

**Table 4:** Pigmented content of maize in the presence and absence of *Bacillus thuringiensis* PM25 under salinity stress.

**NaCl (mM)**	***B. thuringiensis*** **PM25**	**Chl** ***a*** **(mg/g FW)**	**Chl** ***b*** **(mg/g FW)**	**Total Chl (mg/g FW)**	**Carotenoids (mg/g FW)**
0 mM	–PM25	13.63 ± 0.58^bcd^	6.16 ± 0.48^bcd^	19.8 ± 1.06^bcd^	6.67 ± 0.35^bcd^
	+PM25	20.27 ± 1.19^a^	11.23 ± 0.80^a^	31.51 ± 1.75^a^	11.36 ± 0.66^a^
300 mM	–PM25	11.38 ± 0.46^cd^	5.32 ± 0.39^cd^	16.70 ± 0.84^cd^	5.96 ± 0.47^cde^
	+PM25	17.16 ± 0.93^ab^	9.50 ± 0.61^ab^	26.67 ± 1.07^ab^	9.47 ± 0.66^ab^
600 mM	–PM25	9.75 ± 0.92^d^	4.4 ± 0.46^cd^	14.15 ± 1.15^d^	4.10 ± 0.54^de^
	+PM25	15.53 ± 1.17^abc^	8.25 ± 0.66^abc^	23.79 ± 1.83^abc^	8.19 ± 0.55^abc^
900 mM	–PM25	8.60 ± 0.56^d^	2.95 ± 0.45^d^	11.56 ± 1.01^d^	3.25 ± 0.39^e^
	+PM25	12.86 ± 0.80^bcd^	7.02 ± 0.93^bcd^	19.89 ± 1.73^bcd^	6.86 ± 0.40^bcd^

*Growth was measured at 21 days after seed owing under different salt concentration regimes. The treatments exhibit dissimilar letters within rows that represent a significant (p ≤ 0.05) level*.

### Antioxidant enzymatic assays

**Figure 4:**
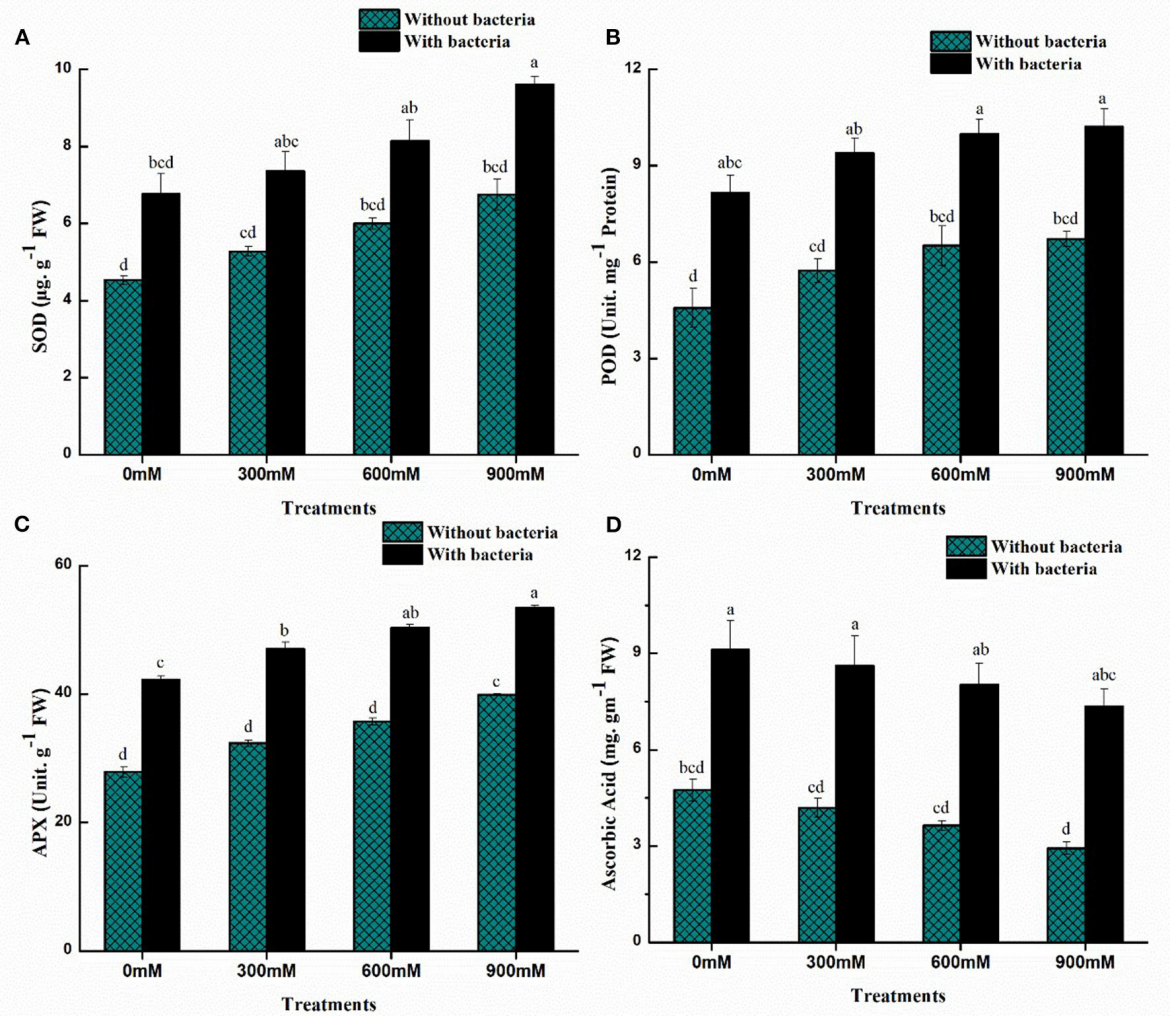
Effects of *Bacillus thuringiensis* PM25 on levels of enzymatic and non-enzymatic antioxidants: **(A)** APX **(B)** POD **(C)** SOD **(D)** Ascorbic Acid. Bars sharing different letter (s) for each parameter are significantly different from each other according to the LSD test (*p* ≤ 0.05). All the data represented are the average of three replications (*n* = 3). Error bars represent the standard errors (SE) of three replicates.

Antioxidants help to reduce oxidative damage by decreasing the formation of reactive oxygen species (ROS) under saline soil. Oxidation of membrane proteins, lipids, or DNA is prevented by scavenging enzymes, including catalase, superoxide dismutase, and ascorbate peroxidase. Under salinity stress, the antioxidant defense system was analyzed. There was an increase in enzymatic antioxidants while a decrease in non-enzymatic antioxidants with increasing salinity stress (Figure 4). In comparison with uninoculated control plants, maize plants with *B. thuringiensis* PM25 accumulated more of these enzymes (APX: 25–34%; POD: 34–44%; SOD: 17–33%) at all concentrations (0, 300, 600, and 900 mM NaCl) (Figure 4). Ascorbic acid content declined as salinity stress increased, but inoculation of *B. thuringiensis* PM25 resulted in a considerable increment (48-60%) in AsA concentration under salinity while comparing to uninoculated maize plants (Figure 4).

### Soluble sugars and protein content of maize

**Figure 5:**
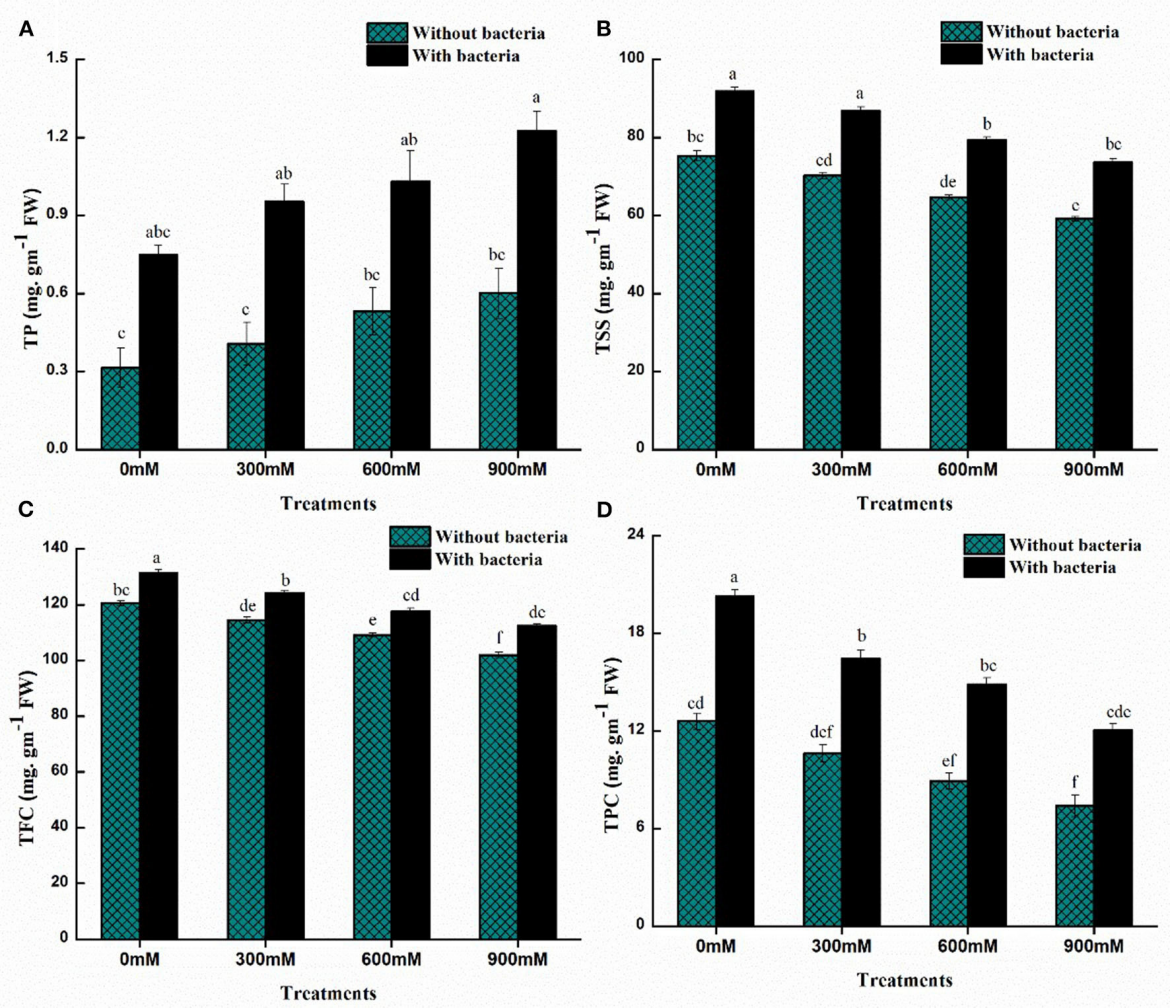
Effects of *Bacillus thuringiensis* PM25 on **(A)** Total soluble sugars **(B)** Protein content **(C)** Flavonoid content **(D)** Phenolic content. Bars sharing different letter (s) for each parameter are significantly different from each other according to the LSD test (*p* ≤ 0.05). All the data represented are the average of three replications (*n* = 3). Error bars represent the standard errors (SE) of three replicates.

Plants protect themselves from salinity and drought stress by accumulating compatible solutes, such as sugars and proteins, to osmotically adjust themselves. The maize plants showed a significant decrease in total soluble sugars (TSS) and a significant increase in total protein content under salinity stress while compared to controlled plants (Figure 5). As 900-mM-NaCl stress was applied, the highest decrease in TSS and a considerable increase in total protein content were recorded compared to the control plants. Furthermore, PM25-treated plants had increased levels of total soluble sugars and proteins compared to uninoculated plants (Figure 5). The application of *B. thuringiensis* PM25 in maize plants significantly enhanced total soluble sugars (18–20%) and total protein content (49–59%) under salinity stress while compared to respective controls (Figure 5).

### Total flavonoids and phenolic content

The effects of *B. thuringiensis* PM25 on the levels of flavonoids and phenolic content of maize plants were studied (Figure 5). The Flavonoids and phenolic content were reduced under salinity stress while compared to controlled plants. A significant decrement in flavonoid and phenolic contents was recorded at 900 mM NaCl (Figure 5). On the other hand, by the inoculation of *B. thuringiensis* PM25, *Zea mays* L. plants proclaimed significant enhancement in total flavonoid (9.24%) and phenolic content (39%) at 900 mM NaCl as compared to uninoculated plants (Figure 5).

### Estimation of oxidative burst and osmolytes

The PGPR aids to alleviate salinity stress in plants by reducing oxidative burst (electrolyte leakage, H_2_O_2_, and MDA content) and accumulating osmolytes (OS) (e.g., free amino acids, glycine betaine, and proline). Oxidative stress indicators [electrolyte leakage (ELL), hydrogen peroxide (H_2_O_2_), and malondialdehyde content (MDA)] and osmolyte [Free amino acids (FAA), glycine betaine (GB), and Proline] levels were greatly influenced by salinity (Tables 5, 6). While comparing to the control plants, the oxidative stress markers (ELL, H_2_O_2_, and MDA content) and osmolytes (FAA, GB, and Proline) in maize plants increased significantly under increasing salinity stress (Tables 5, 6). A significant increase was also observed in oxidative stress markers at 900-mM-salinity stress (Table 5). On the other hand, electrolyte leakage, hydrogen peroxide, and malondialdehyde content were significantly reduced (Table 5), while levels of compatible solutes were increased in bacterial inoculated plants while compared to uninoculated ones (Table 6). The application of *B. thuringiensis* PM25-inoculated maize plants showed a characteristic reduction in ELL (14–19%), H_2_O_2_ (28–54%), and MDA content (9–18%) (Table 5), while significant elevation in levels of FAA (42–47%), GB (45–52%), and proline (30–36%) under salinity stress as compared to respective controls (Table 6).

**Table 5:** Level of oxidative stress markers in the presence and absence of *Bacillus thuringiensis* PM25 under salinity stress.

**NaCl (mM)**	***B. thuringiensis*** **PM25**	**ELL (%)**	**H**_2_**O**_2_ **(**μ**mol/g FW)**	**MDA (nmol/g FW)**
0 mM	–PM25	36.06 ± 0.68^cd^	36.17 ± 0.72^de^	21.06 ± 0.67^bc^
	+PM25	29.23 ± 0.68^e^	33.09 ± 1.06^e^	9.75 ± 1.10^d^
300 mM	–PM25	41 ± 0.79^c^	41.58 ± 1.61^cd^	23.21 ± 0.77^b^
	+PM25	33.06 ± 1.01^de^	36.8 ± 0.66^de^	12.61 ± 0.66^d^
600 mM	–PM25	48.43 ± 0.87^b^	49.05 ± 1.09^ab^	26.02 ± 0.60^ab^
	+PM25	39.4 ± 1.20^c^	42.29 ± 1.25^bcd^	15.23 ± 0.80^cd^
900 mM	–PM25	54.76 ± 0.54^a^	55.3 ± 0.96^a^	29.57 ± 1.12^a^
	+PM25	46.9 ± 0.46^b^	45.23 ± 1.19^bc^	21.26 ± 1.34^b^

*Growth was measured at 21 days after seed owing under different salt concentration regimes. ELL–Electrolyte leakage, H_2_O_2_-Hydrogen peroxide, MDA–Malondialdehyde. The treatments exhibit dissimilar letters within rows that represent a significant (p ≤ 0.05) level*.

**Table 6:** Level of osmolytes in the presence and absence of *Bacillus thuringiensis* PM25 under salinity stress.

**NaCl (mM)**	***B. thuringiensis*** **PM25**	**Free amino acid (mg/g DW)**	**Glycine betaine (**μ**g/g DW)**	**Proline (**μ**mol/g FW)**
0 mM	–PM25	15.72 ± 0.58^e^	10.68 ± 0.97^f^	50.48 ± 1.59^f^
	+PM25	27.76 ± 0.85^bc^	22.18 ± 0.68^cd^	77.76 ± 0.78^c^
300 mM	–PM25	18.97 ± 0.93^de^	13.82 ± 0.77^ef^	53.54 ± 1.51^ef^
	+PM25	32.83 ± 0.68^b^	25.61 ± 0.39^bc^	83.44 ± 0.66^bc^
600 mM	–PM25	21.25 ± 1.05^de^	15.77 ± 0.97^e^	57.07 ± 0.80^e^
	+PM25	39.83 ± 1.33^a^	28.92 ± 0.54^ab^	87.93 ± 0.36^ab^
900 mM	–PM25	24.42 ± 0.78^cd^	17.44 ± 0.85^de^	65.19 ± 0.81^d^
	+PM25	44.08 ± 1.06^a^	32.48 ± 0.67^a^	93.37 ± 0.61^a^

*Growth was measured at 21 days after seed owing under different salt concentration regimes. The treatments exhibit dissimilar letters within rows that represent a significant (p ≤ 0.05) level*.

### Stress-related gene amplification

The multi-stress-related genes were amplified in *B. thuringiensis* PM25. The universal primers were used for PCR amplification of the *ItuC* (506 bp), *sfp* (675 bp), and *srfAA* (268 bp) genes of halo-tolerant *B. thuringiensis* PM25 and exhibited sharp bands at respective base pair (bp) (Supplementary material 1).

### Expression level of antioxidant (APX and SOD) genes

**Figure 6:**
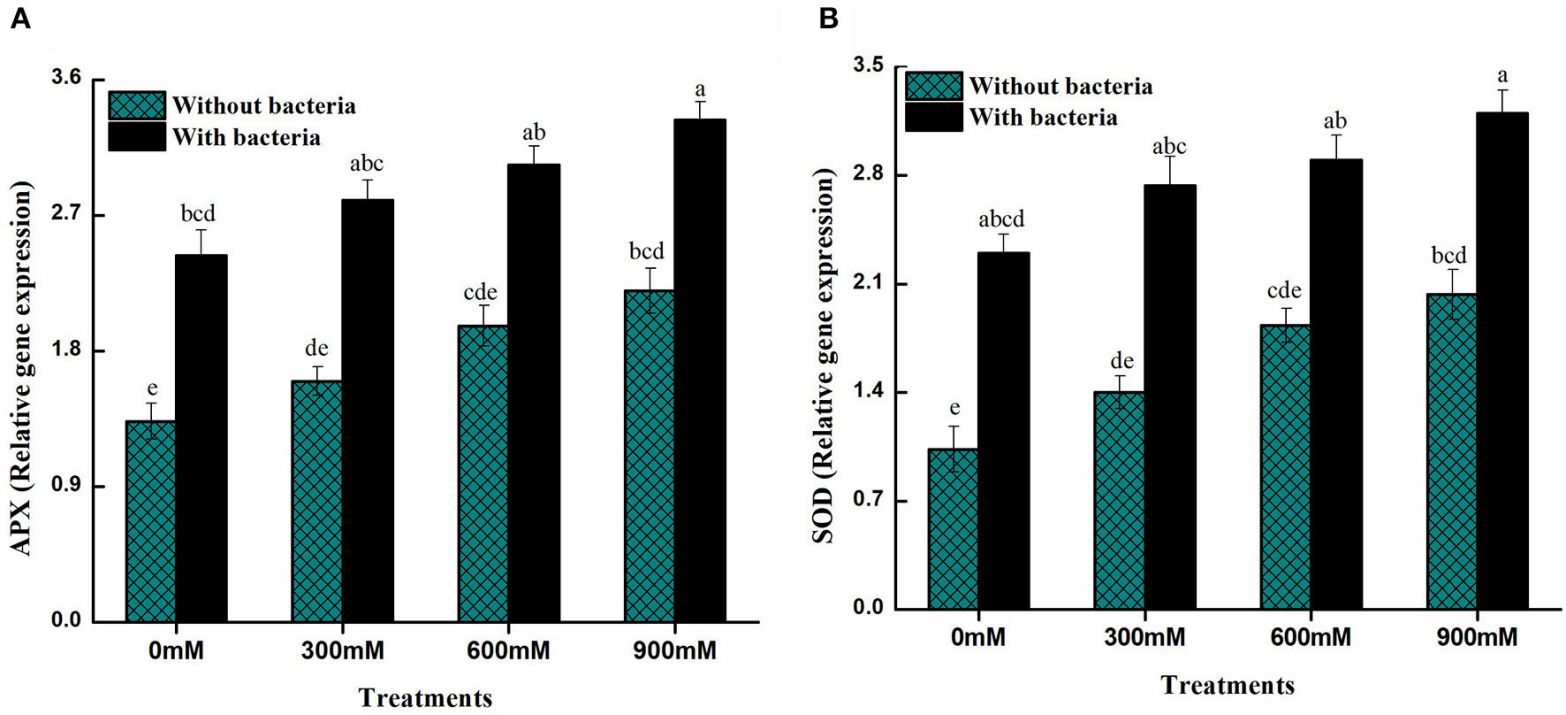
Expression levels of antioxidant genes of maize in the absence and presence of *B. thuringiensis* PM25 under salinity stress **(A)** Ascorbate peroxidase (APX) **(B)** Superoxide dismutase (SOD). Bars sharing different letter (s) for each parameter are significantly different from each other according to the LSD test (*p* ≤ 0.05). All the data represented are the average of three replications (*n* = 3). Error bars represent the standard errors (SE) of three replicates.

Plants inoculated with PGPB under high salt conditions produces antioxidant genes that are involved in maintaining levels of ROS and confirm the role of PGPB in free radicals scavenging under salinity stress condition. Under control conditions, *B. thuringiensis* PM25 inoculation enhanced the expression of the two antioxidant genes (APX and SOD) in relation to uninoculated controls (Figure 6). Furthermore, *B. thuringiensis* PM25-inoculated plants revealed a higher expression level of antioxidant genes compared to uninoculated maize plants under salinity stress (Figure 6).

### Principal component and Pearson correlation analysis

**Figure 7:**
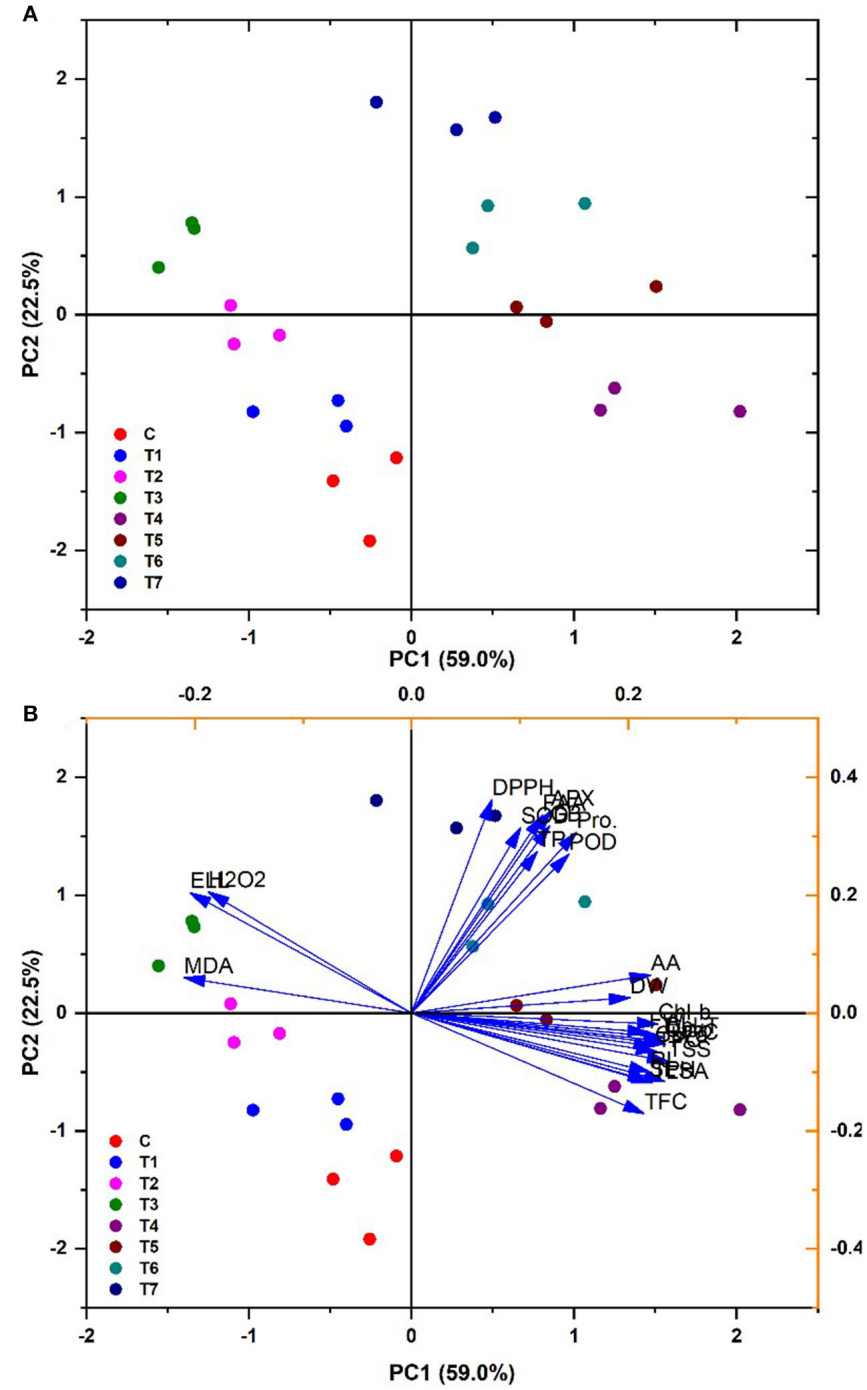
PCA biplot showing the categorization of PM25 based on its effects on maize growth-promoting characteristics under salinity stress **(A)** Cluster analysis **(B)** PCA Biplot analysis.

There was a correlation among multiple variables under salinity regimes and *B. thuringiensis* PM25 treatment in the principal component Biplot analysis. Significantly correlated variables were grouped in quadrates. Biplot indicated an 81.5% variance (PC_1_ = 59%; PC_2_ = 22.5%) (Figure 7). There was a positive correlation between antioxidants, agro-morphological traits, pigmented content, relative water content, and compatible solutes of maize. The antioxidant activity of leaves (DPPH) and oxidative stress indices were adversely interrelated with others.

**Figure 8:**
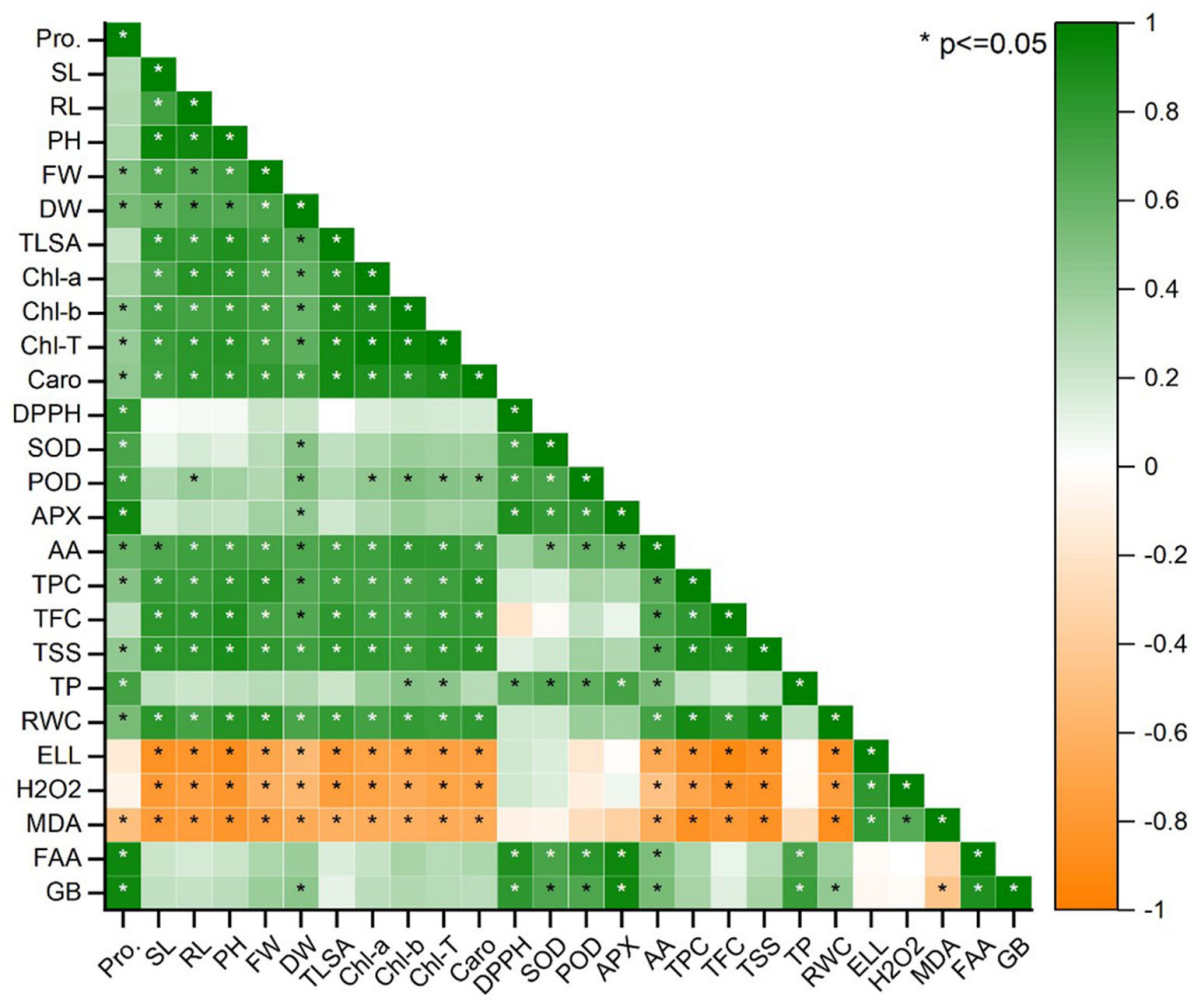
Pearson correlation between antioxidants and biochemical traits with plant biomass parameters under salinity stress; Pro, (Proline), SL (Shoot length), RL (Root length), PH (Plant height), FW (Fresh weight), DW (Dry weight), LA (Leaf area), Chl a (Chlorophyll a), Chl b (Chlorophyll b), T. Chl (Total chlorophyll), Caro (Carotenoids), DPPH (Radical scavenging capacity), SOD (Superoxide dismutase), POD (Peroxidases), APX (Ascorbate peroxidase), AA (Ascorbic acid), TPC (Total phenolic content), TFC (Total flavonoid content), TSS (Total soluble sugars), TP (Total Protein), RWC (Relative water content), EL (Electrolyte leakage), H_2_O_2_ (Hydrogen peroxide), MDA (Malondialdehyde), FAA (Free amino acids), GB (Glycine betaine). The treatments exhibit (*) within rows that represent a significance (*p* ≤ 0.05) level.

Plant biomass was examined using Pearson's correlation of antioxidants and biochemical characteristics (Figure 8). In maize plants, pigmented content was found to have a substantial productive correlation with SL, FW, and RL. Increasing these features is significantly interrelated to a dramatic rise in plant biomass production (Figure 8). Total soluble sugar, pigmented content, RWC, total phenolic content, and carotenoids were all strongly related to SL, FW, and RL. The increment in biomass (SL, RL, and FW) and pigmented content resulted in elevated levels of FAA, SOD, APX, GB, TP, POD, and proline. All plant biomass parameters had a high negative connection with H_2_O_2_ MDA, EL, and DPPH. On the other hand, electrolyte leakage, total soluble sugars total flavonoid content, the antioxidants, and radical scavenging capacity glycine betaine, total protein, hydrogen peroxide, total phenolic content, free amino acids, malondialdehyde, antioxidant enzymes, and radical scavenging capacity were found to have a strong negative relationship. Under various treatments, lowering antioxidants causes a decrease in plant biomass (Figure 8).

## Discussion

Sustainable agriculture needs crop yield and quality while ameliorating abiotic constraints (Amna et al., 2021). Nowadays, food security is a critical issue owing to the increasing population (Fróna et al., 2019). This can be accomplished by successfully introducing putative abiotic stress tolerance and PGPR. Keeping this in mind, the current work aims to investigate the potential of halo-tolerant *Bacillus thuringiensis* PM25 on maize growth promotion and salinity stress alleviation.

The potential of halo-tolerant *B. thuringiensis* PM25 against salinity stress was tested in this study. This halo-tolerant *B. thuringiensis* PM25 showed strong resilience to salinity (3 M NaCl) stress (Figure 1). The bacterial survival was steadily reduced under salinity stress (Figure 2A), which correlates with previous results (Ali et al., 2022b). The bacterial population was reduced due to osmotic and water imbalance caused by salinity (Ali et al., 2022a).

Under salinity stress, the halo-tolerant *B. thuringiensis* PM25 developed much more floc production, biofilm, and Na^+^ absorption (Figures 2B–D). Increased EPS-producing bacteria availability improves food and water uptake in the rhizosphere (Kalam et al., 2020). By raising water content in plants, the EPS can lessen the detrimental effects of osmotic stress and, hence, increase the plant's biomass (Ghosh et al., 2019). As a barrier between the cells and salinity stress, biofilm protects bacterial cells inside the EPS layer. Due to EPS production, bacteria uptake Na^+^ while retaining a high population. The EPS-producing bacteria in this study reduced the amount of available Na^+^ in the soil, hence, mitigating saline stress. The UPMRB9 produced a lot of EPS and biofilm at 1.5-M salinity (Shultana et al., 2020). Hong et al. (2017), reported that the adhesive qualities of EPS helped bacteria combine with soil particles and Na^+^, lowering salt toxicity in plants. According to Basu et al. (2021), bacteriological polysaccharides are acidic and have a strong attraction for specific ions. A higher level of NaCl increased biofilm development in *Bacillus* spp., according to Kasim et al. (2016), with the largest peak occurring at 500 mM NaCl.

Under salinity stress, the PGPR, *B. thuringiensis* PM25 produced considerable amounts of IAA, siderophore, ACC deaminase, and EPS (Figure 3). Indole acetic acid (IAA) aids seed germination and the formation of longer roots with more root hairs, which aids nutrient intake indirectly. It also has a lot of potential for large-scale cereal crop production (Khan et al., 2016; Aloo et al., 2019; Bensidhoum and Nabti, 2019). It has been claimed that IAA produced by bacteria aids root growth by inducing cell elongation and/or responding to cell division (Vimal et al., 2019). The IAA production is linked to root development and structural alterations in response to stress (Abbas et al., 2018). Previous research found that PGPB synthesis of IAA increased plant growth in *Bacillus* sp., *Enterobacter* sp., *Bacillus thuringiensis, Bacillus tequilensis*, and *Bacillus mycoides* (Guerrero-Barajas et al., 2020).

Under salinity stress, *B. thuringiensis* PM25 followed a similar pattern as IAA (Figure 3) and enhanced plant growth by siderophore production. The presence of siderophore-producing PGPR in the rhizosphere can minimize the effect of salinity on Fe availability in the soil (Ferreira et al., 2019). Plants acquired iron from the soil and used siderophore-producing bacteria to suppress phytopathogens. Plants' chlorophyll biosynthesis, photosynthetic electron transport, and respiratory chain functioning were all influenced by iron chelation (Saha et al., 2016). The iron chelation increased the production and repair of DNA and RNA (Ferreira et al., 2019). During plant growth, many siderophore-producing endophytic bacterial communities, such as *Sphingomonas, Pseudomonas, Enterobacter*, and *Burkholderia* alternated (Ferrarezi et al., 2022).

Under salinity stress, *B. thuringiensis* PM25 degraded ACC into ammonia and α-ketobutyrate in roots, redirected ethylene production routes, and limited ethylene biosynthesis (Figure 3). Another technique for dealing with salt stress is for rhizospheric bacteria to have ACC deaminase activity (Gamalero and Glick, 2015). Under saline/sodic stress, ethylene production increased, inhibiting seed germination and plant growth (Koevoets et al., 2016). However, recruitment of ACC deaminase-producing bacteria to seedling roots metabolized ACC into ammonia and α-ketobutyrate, redirecting ethylene production pathways. As a result, the negative effects of ethylene are mitigated (Carlos et al., 2016; Sarkar et al., 2018), and germination is improved, leading to better plant growth under saline conditions (Kumari et al., 2018; Marag and Suman, 2018).

Some plant growth-promoting rhizobacteria (PGPR) produce exopolysaccharides (EPS) or surface polysaccharides. Under salinity stress, *B. thuringiensis* PM25 produced significant EPS while compared to control (Figure 3). The EPS-producing PGPR strains increase soil salinity tolerance, improve soybean (*Glycine maxima*) plant growth (Zhang et al., 2022), and restrict Na^+^ absorption by wheat roots (Souza et al., 2015). These EPS aids in the formation of bacterial aggregates, which enhances soil aeration, water infiltration, and root growth (Bhise and Dandge, 2019).

Salinity is one of the most significant abiotic constraints in agricultural production, particularly in dry and semi-arid regions. It reduces the amount of water available to plants, which has disastrous consequences for both growth and productivity (Hussain et al., 2019). Our results showed that the *B. thuringiensis* PM25 used in the current study promoted the vegetative growth of maize plants. It was worth mentioning that *B. thuringiensis* PM25 improved agro-morphological traits compared to uninoculated plants (Tables 2, 3). In comparison to control maize plants, PM25-inoculation overcame the unfavorable effects of salinity stress on growth and yield and promoted phenotypic traits of maize under salinity stress (Tables 2, 3). Inoculating wheat plants with PGPB increased the agro-morphological features in a pot experiment in saline soil (Paredes-Páliz et al., 2016; Bakka and Challabathula, 2020). Plant growth-promoting rhizobacteria (PGPR) modulate root architecture by establishing extended rhizo-sheaths, preventing salt uptake *via* roots by confining Na^+^ ions in the EPS matrix, assisting the plant in maintaining ionic equilibrium. The increased growth of inoculated plants under a controlled and stressful environment is most likely due to IAA synthesis by the PGPR (Abbas et al., 2018).

Salinity causes osmotic stress, one of the most evident signs of a drop in leaf relative to water (Silambarasan et al., 2019). Under salinity stress, bacterial-inoculated maize plants had a greater RWC than the controls (Table 3)). PGPR significantly increases the roots and development of salt-stressed plants by boosting water usage efficiency and improving the RWC (Singh and Jha, 2016; Nosheen et al., 2021).

Our results demonstrated that co-inoculation of *B. thuringiensis* PM25 augmented maize plants' radical scavenging potential (Table 3). Increased ROS scavenging ability has previously been reported in canola (Li et al., 2017), rice (Sarkar et al., 2018), chickpea (El-Esawi et al., 2019), sunflower (Gupta et al., 2019), and lettuce plants (Silambarasan et al., 2019) under abiotic stress by inoculating a plant with PGP microorganisms.

The reduction in plant growth was based on an increase in Na^+^ ion levels and oxidative stress, which reduced photosynthetic efficiency, ion imbalance, and membrane integrity (Desoky et al., 2020). Inoculating maize with *B. thuringiensis* PM25 overcomes the negative impacts of salinity stress and increased physiological attributes, such as chlorophyll content (Table 4). Our findings backed up a prior study that found that PGPB inoculation increased chlorophyll synthesis and accelerated radish plant development (Al Kahtani et al., 2021). Tomato plants infected with PGPB, *Achromobacter piechaudii* ARV8, increased photosynthetic activity in salinity-stressed plants (Shrivastava and Kumar, 2015). According to Enebe and Babalola (2018), PGPR assists in the stability of photosynthetic pigments.

Low molecular weight antioxidants are produced by plants to impart salinity stress tolerance (Xu et al., 2015). Under salinity stress, inoculating maize plants with *B. thuringiensis* PM25 significantly enhanced enzymatic (APX, POD, SOD) and non-enzymatic (Ascorbic acid) antioxidants (Figure 4). Antioxidant activity was increased by the application of PGPB (Hashem et al., 2016). Furthermore, 5-aminolevulinic acid-producing bacteria reduced H_2_O_2_ production and boosted APX, POD, and SOD antioxidant activities in salinity-stressed rice (Kantachote et al., 2016; Wu et al., 2018). Ascorbic acid (AsA) is an excellent ROS scavenger due to its ability to activate enzymatic and non-enzymatic processes that influence H_2_O_2_ and maintain cell membranes (Akram et al., 2017).

For instance, total protein content (TP) and total soluble sugars (TSS) in plants under saline stress are important defensive strategies to cope with salt stress. Under salinity stress, the inoculation of halo-tolerant *B. thuringiensis* PM25 increased total soluble sugars and protein content (Figure 5). Plants inoculated with PGPR could increase the production of soluble proteins and soluble carbohydrates in high-salinity environments, allowing them to withstand oxidative and osmotic stressors (Qu et al., 2016; Bhise and Dandge, 2019). Previous studies have also shown that inoculating wheat seeds with the *Bacillus aquimaris* strain increases soluble sugars in saline circumstances, improving plants' growth and biomass (Upadhyay and Singh, 2015).

Under salinity stress, *B. thuringiensis* PM25 showed a considerable increase in flavonoid and polyphenol content (Figure 5). For instance (Sofy et al., 2021) discovered that inoculating pea plants with PGPB increases the phenolic content to reduce salt stress. Under salinity stress, the significant increase of flavonoids and phenolic content in halo-tolerant PGPB-treated plants may help in the inactivation of ROS and the breakdown of H_2_O_2_ to alleviate oxidative stress (Gago et al., 2016).

In this study, electrolyte leakage (ELL) and ROS levels (MDA and H_2_O_2_ content) were increased under salinity stress (Table 5). However, inoculation with PGPR (*B. thuringiensis* PM25) decreases the ROS levels in maize plants (Table 5). Likewise, maize inoculated with PGPR remarkably declined the ELL, MDA, and H_2_O_2_ levels under NaCl stress conditions (El-Esawi et al., 2018). Plant beneficial microbes of the rhizosphere can help to improve the integrity of plant cell membrane by inhibiting oxidative damage (Abadi and Sepehri, 2016). These findings are consistent with (Pinedo et al., 2015; Abd Allah et al., 2018), in which PGPB-inoculated maize and white clover plants lowered oxidative stress indicators under salinity.

Salinity stress raised the level of osmolytes considerably (Table 6). The present findings showed that PGPR inoculation (*B. thuringiensis* PM25) increased the levels of osmolytes under salinity stress (Table 6). Compatible organic solutes regulate leaf water potential and protect plants against osmotic imbalance (Upadhyay and Singh, 2015; Iqbal et al., 2016). Wheat treated with PGPR maintains growth under saline stress by accumulating compatible solutes (Xu et al., 2017). PGPR application significantly enhanced wheat yield under saline conditions (Akhtar et al., 2015; Farooq et al., 2015; Naili et al., 2018).

In the current investigation, surfactant-producing *sfp* and *srfAA* and while Iturin C (*ItuC*) gene, which encodes antibiotic synthesis, was amplified in *B. thuringiensis* PM25 (Supplementary material 1). The bio-surfactants improve colonization and yield by strengthening the interface between rhizobacteria that promote plant growth and roots (Khare and Arora, 2021; Kumar et al., 2021). Because the bacterial strain improved maize's growth and tolerance to salinity, this bacteria can be used as a bio-fertilizer and multi-stress resistant substance for plants.

Furthermore, inoculating *B. thuringiensis* PM25 massively improved the gene expression associated with salt tolerance and antioxidant enzymes (Figure 6). By regulating SOD and APX genes, salinity stress was alleviated in chickpeas (Elkelish et al., 2019). Salinity stress was mitigated in rice seedlings where levels of antioxidants were increased by inoculating PGPB (Ji et al., 2020).

## Conclusion

In the recent scenario of the increasing global population and changing climatic conditions, sustainable agricultural methods are of immediate need. The current study found that inoculating maize plants with halo-tolerant *B. thuringiensis* PM25 enhanced their resistance to salinity stress. The treatment of *B. thuringiensis* PM25 substantially alleviates salinity stress. This halo-tolerant bacteria *B. thuringiensis* PM25 has plant growth-promoting features and, in liquid formulations, it stimulated maize growth and biomass under salinity stress. This rhizospheric *B. thuringiensis* PM25 was critical in relieving salinity stress in maize plants by modifying the antioxidant defense system, lowering oxidative burst, and accumulating compatible solutes. According to the findings of the current study, halo-tolerant *B. thuringiensis* PM25 has the potential to be used as a viable alternative and environmentally acceptable for facilitating maize production and mitigating salinity stress. A pot experiment with *B. thuringiensis* PM25-inoculation at various salinity stress gives a baseline for analyzing potential PGPR under salinity stress. However, more study on natural saline soil under field circumstances is necessary. The use of PGPR in the agriculture field is a green option to increase productivity and crop yield.

## Data Availability Statement

The original contributions presented in the study are included in the article/Supplementary material, further inquiries can be directed to the corresponding authors.

## Author contributions

AH and BA: conceptualization. TD, KA, and MJ: data curation. AH, SA, S, and MA: formal analysis. RM, CM, TD, and KA: funding acquisition. S, RM, CM, AH, and MJ: software. BA and AH: investigation and methodology. BA, AH, and SA: writing—original draft. BA, AH, SA, MJ, S, MA, TD, KA, CM, RM, SS, and DA: writing—review and editing. AH, BA, TD, KA, RM, SS, DA, and CM: visualization. TD and KA: validation. All authors approved the final version of the manuscript.

## Funding

The authors extend their appreciation to the Researchers supporting project number (RSP-2021/197), King Saud University, Riyadh, Saudi Arabia. The publication was supported by funds from the National Research Development Projects to finance excellence (PFE)-14/2022-2024 granted by the Romanian Ministry of Research and Innovation.

## Conflict of interest

The authors declare that the research was conducted in the absence of any commercial or financial relationships that could be construed as a potential conflict of interest.

## Publisher's note

All claims expressed in this article are solely those of the authors and do not necessarily represent those of their affiliated organizations, or those of the publisher, the editors and the reviewers. Any product that may be evaluated in this article, or claim that may be made by its manufacturer, is not guaranteed or endorsed by the publisher.
