# Supplementary material for: Bacillus thuringiensis PM25 ameliorates oxidative damage of salinity stress in maize via regulating growth, leaf pigments, antioxidant defense system, and stress responsive gene expression
Source: Front Plant Sci. 2022 Jul 28;13:921668. doi: 10.3389/fpls.2022.921668 (PMC9366557; doi:10.3389/fpls.2022.921668)
Supplement: Supplementary file 1 [file Data_Sheet_1.docx]

**Table S1. Experimental design for pot studies**

| **Treatments** | **Description** |
| --- | --- |
| T0 | Control |
| T1 | 300 mM NaCl |
| T2 | 600 mM NaCl |
| T3 | 900 mM NaCl |
| T4 | *B. thuringiensis* PM25 |
| T5 | PM25 + 300 mM |
| T6 | PM25 + 600 mM |
| T7 | PM25 + 900 mM |

**Table S2. Primers Used for Gene Amplifications**

| **Genes** | **Primers** | **Primer Sequence (5′-3′)** | **PCR Product Size Expected/Detected (bp)** |
| --- | --- | --- | --- |
| *ItuC* | *ITUC-F1*  *ITUC-R1* | 5′-CCCCCTCGG TCAAGTGAATA-3′  5′-TTGGTTAAG CCCTGATGCTC-3′ | 506 |
| *sfp* | *sfp F*  *sfp R* | 5′ATGAAGATTTACGGAATTTA-3′  5′-TTATAAAAGCTCTTCGTACG-3′ | 675 |
| *srfAA* | *srfAA F*  *srfAA R* | F-5′-TCGGGACAGGAAGACATCAT-3′  R-5′-CCACTCAAACGGATAATCCTGA-3′ | 268 |

**Table S3.** **PCR recipe for real-time analysis.**

| **Component** | **Volume (per Reaction)** |
| --- | --- |
| Sterile ddH_2_O | 3 μL |
| Primer (forward + reverse) 10mM | 2 μL |
| SYBR Green I Master (Roche) 2X | 10 μL |
| cDNA Template | 5 μL |
| **Total Volume** | **20 μL** |


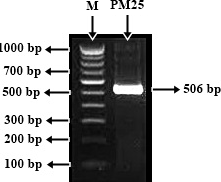

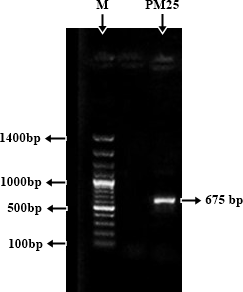

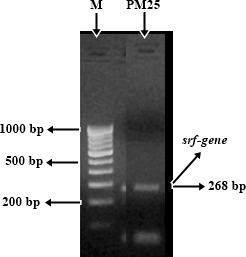


**Supplementary 1.** Amplification of abiotic stresses related genes: (a) *ItuC-gene* (b) *sfp-gene* (c) *srfAA- gene*. (M) represents marker.
